# Hypoxia-Inducible Histone Lysine Demethylases: Impact on the Aging Process and Age-Related Diseases

**DOI:** 10.14336/AD.2015.0929

**Published:** 2016-03-15

**Authors:** Antero Salminen, Kai Kaarniranta, Anu Kauppinen

**Affiliations:** 1 Department of Neurology, Institute of Clinical Medicine, University of Eastern Finland, Kuopio, Finland; 2 Department of Ophthalmology, Institute of Clinical Medicine, University of Eastern Finland, Kuopio, Finland; 3 Department of Ophthalmology, Kuopio University Hospital, Finland; 4 School of Pharmacy, Faculty of Health Sciences, University of Eastern Finland, Kuopio, Finland

**Keywords:** Aging, Chromatin, Epigenetic, Hypoxia, Pseudohypoxia, Senescence

## Abstract

Hypoxia is an environmental stress at high altitude and underground conditions but it is also present in many chronic age-related diseases, where blood flow into tissues is impaired. The oxygen-sensing system stimulates gene expression protecting tissues against hypoxic insults. Hypoxia stabilizes the expression of hypoxia-inducible transcription factor-1α (HIF-1α), which controls the expression of hundreds of survival genes related to e.g. enhanced energy metabolism and autophagy. Moreover, many stress-related signaling mechanisms, such as oxidative stress and energy metabolic disturbances, as well as the signaling cascades via ceramide, mTOR, NF-κB, and TGF-β pathways, can also induce the expression of HIF-1α protein to facilitate cell survival in normoxia. Hypoxia is linked to prominent epigenetic changes in chromatin landscape. Screening studies have indicated that the stabilization of HIF-1α increases the expression of distinct histone lysine demethylases (KDM). HIF-1α stimulates the expression of KDM3A, KDM4B, KDM4C, and KDM6B, which enhance gene transcription by demethylating H3K9 and H3K27 sites (repressive epigenetic marks). In addition, HIF-1α induces the expression of KDM2B and KDM5B, which repress transcription by demethylating H3K4me2,3 sites (activating marks). Hypoxia-inducible KDMs support locally the gene transcription induced by HIF-1α, although they can also control genome-wide chromatin landscape, especially KDMs which demethylate H3K9 and H3K27 sites. These epigenetic marks have important role in the control of heterochromatin segments and 3D folding of chromosomes, as well as the genetic loci regulating cell type commitment, proliferation, and cellular senescence, e.g. the INK4 box. A chronic stimulation of HIF-1α can provoke tissue fibrosis and cellular senescence, which both are increasingly present with aging and age-related diseases. We will review the regulation of HIF-1α-dependent induction of KDMs and clarify their role in pathological processes emphasizing that long-term stress-related insults can impair the maintenance of chromatin landscape and provoke cellular senescence and tissue fibrosis associated with aging and age-related diseases.

Ever since the Cambrian period, oxygen availability has been in the center of energy metabolism and thus exposure to hypoxia or ischemia can jeopardize the maintenance of tissue homeostasis. During evolution, organisms have evolved mechanisms to survive in hypoxia by developing the oxygen-sensing systems, which can induce gene expression profiles protecting against hypoxia-induced injuries. In metazoans, the hypoxia response is induced by the oxygen-dependent control of prolyl 4-hydroxylases (PHD1-3), which regulate the cellular expression of hypoxia-inducible transcription factors (HIF1-3) [1-3]. The PHD-HIF signaling is stimulated by the deficiency of oxygen, which inhibits the activity of PHDs preventing the hydroxylation of HIF factors and thus blocking their subsequent degradation, i.e. hypoxia stabilizes the cellular expression of HIF transcription factors. The HIF-induced hypoxia response involves the expression of hundreds of survival genes, e.g. enhancing energy metabolism and autophagy. Remarkably, hypoxia is not the only insult that can stabilize the expression of HIF factors. There are several other signals, mostly related to stresses, which can increase the expression of HIF factors and thus improve cellular survival (see below) (Fig. 1). However, a chronic activation of HIF factors can have detrimental effects, e.g. stimulate cellular senescence and tissue fibrosis commonly enhanced in age-related diseases.

A substantial literature indicates that hypoxia is linked to the epigenetic modification of chromatin landscape. Interestingly, the stabilization of HIF-1α increases the expression of several histone lysine demethylases (KDM), which are crucial enzymes in the control of gene expression in hypoxia but in collaboration with other chromatin modifiers they can also affect heterochromatin structures, genome stability, and reprogramming of cellular senescence loci (see below). Increase in cellular senescence is associated with the aging process and many age-related diseases. We will review the molecular aspects of the HIF-1α-induced expression of KDMs and clarify their role in the epigenetic modifications associated with the aging process and the appearance of age-related diseases. We propose that a chronic induction of HIF-1α, especially through stress-related signals, can impair the regulation of chromatin landscape and thus provoke cellular senescence and aggravate age-related diseases.

**Figure 1.**
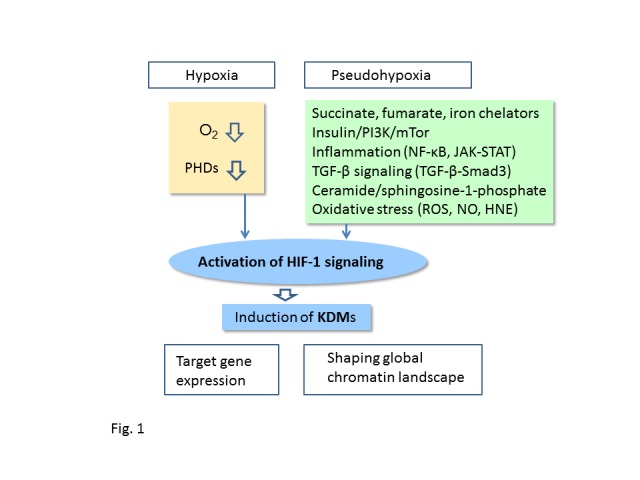
**Induction of KDM expression by HIF-1α signaling**. HIF-1α signaling can be activated by hypoxia and several stress-related signaling pathways, commonly called pseudohypoxia since they activate HIF-1α signaling in normoxia. KDMs induced by HIF-1α control the transcription of HIF-1α target genes but in addition, they can modify the global chromatin landscape provoking pathological changes linked to the aging process and age-related diseases. Abbreviations: HIF-1α, hypoxia-inducible factor-1α; HNE, 4-hydroxynonenal; JAK, Janus kinase; KDM, histone lysine demethylase; mTor, mammalian target of rapamycin; NF-κB, nuclear factor-κB; NO, nitric oxide; PI3K, phosphoinositide 3-kinase; ROS, reactive oxygen species; Smad3, SMAD family member 3; STAT, signal transducer and activator of transcription; TGF-β, transforming growth factor-β.

## Hypoxia stimulates HIF-1α signaling

Hypoxia, i.e. a decline in the oxygen partial pressure, is an environmental stress but hypoxia is also commonly present in many pathological conditions in which blood flow into tissues is impaired, e.g. in ischemia/stroke, arteriosclerosis, and inflammatory disorders. Organisms effectively respond to hypoxia, e.g. switching their energy production from oxidative to glycolytic pathway [4, 5]. Moreover, animals can adapt to hypoxic conditions by generating hypoxia resistance [6, 7]. Early 1990, Gregg Semenza and his collaborators discovered that hypoxia induced a nuclear accumulation of DNA-binding factor, which was *de novo* synthesized in several cell lines [8-11]. They called that protein hypoxia-inducible factor-1 (HIF-1). HIF-1 protein was able to bind to the promoter of human *Erythropoietin* (EPO) gene, which is a well-known hypoxia-inducible gene. They also observed that the binding of HIF-1 protein induced the transactivation of *EPO* gene in hypoxia. Subsequently, they cloned the *HIF-1α* gene and identified the HIF-1α protein [11]. At the same time, Peter Ratcliffe and his team clarified the oxygen-regulated, cis-acting enhancer sequences in target genes, such as *EPO* and *LDH-A* genes [12-14]. HIF-1α protein can bind to a specific DNA sequence as a heterodimer with HIF-1β protein, also known as *Aryl hydrocarbon receptor nuclear translocator* (ARNT). This binding site was termed the hypoxia response element (HRE), present at the promoters of hypoxia-inducible genes. Wang et al. [11] observed that HIF-1α protein was post-translationally modified in hypoxia and its post-hypoxic decay was very rapid indicating that HIF-1α protein is unstable in normoxia. In 2001, several studies demonstrated that HIF-1α was hydroxylated by specific prolyl-4-hydroxylases (PHDs) and subsequently ubiquitylated by von Hippel-Lindau E3 ligase (pVHL) [15-17]. This ubiquitylation directed HIF-1α protein to proteasomal degradation. Interestingly, the PHD1-3 enzymes, also called EGLN1-3, are 2-oxoglutarate-dependent dioxygenases (2-OGDOs), which require O_2_ for a substrate and thus they are inactive in hypoxia [5, 17, 18]. This dependency on oxygen prevents the hydroxylation of HIF-1α protein in hypoxia and thus enhances its stabilization and subsequent transactivation of target genes. The 2-OGDO enzymes are not only sensors for oxygen availability but also for the presence of 2-oxoglutarate, a Krebs cycle intermediate, and iron homeostasis [19, 20]. In addition to PHD1-3, the 2-OGDO family includes both DNA and histone demethylases [21, 22], as discussed later. We have recently reviewed the potential role of 2-OGDO enzymes in the control of aging process [23].

The family of HIF factors contain three isoforms, of which HIF-1α and HIF-2α are activating factors and HIF-3α is an inhibitor lacking the C-terminal transactivation domain [24]. There seems to be another negative feedback system preventing the stabilization of HIF-1α protein in hypoxia, since HIF-1α increases the expression of PHD2/3 and thus can reduce the accumulation of HIF-1α protein [25] (Fig. 1). HIF-1α and HIF-2α factors have distinct expression patterns in hypoxia as well as specific target genes although they both bind to the HRE elements [26, 27]. Moreover, they have various post-translational modifications, distinct interactions with other transcription factors, and thus they have clear functional differences. Gene expression profiling studies have indicated that HIF-1α protein controls the expression of about 200-1000 genes, the most common target genes regulate angiogenesis, autophagy, glucose uptake, and glycolytic enzymes, i.e. those genes which increase adaptation to hypoxia [28, 29]. In addition, HIF-1α factors control immune responses, embryonic development, and tumorigenesis [30, 31]. Hypoxia can also regulate gene expression via the generation of miRNAs, denoted hypoxamirs [32]. For instance, it has been observed that HIF-1α induced the expression of miR-210, which subsequently affected mitochondrial metabolism, cell cycle progression, DNA repair, and cancer formation [33]. These examples indicate that HIF-1α factor can stimulate a complex network of genes to facilitate the adaptation to hypoxia and enhance the survival of organisms.

## Pseudohypoxia stimulates HIF-1α signaling in normoxia

Reduced oxygen availability is not the only way to stimulate the HIF-1α signaling. The activation of HIF-1α and subsequent target gene expression under normal oxygen pressure is called pseudohypoxic response (Fig. 1). There are several mechanisms which can induce the HIF-1α signaling in normoxia by e.g. (i) suppressing the activity of PHDs with mitochondrial metabolites, such as succinate and fumarate, as well as nitric oxide (NO) and iron chelators, (ii) inhibiting the VHL ligase-induced ubiquitination of HIF-1α, (iii) stabilizing HIF-1α expression through post-translational modifications, (iv) enhancing the transcription and transcriptional activity of HIF-1α. In 2005, Selak et al. [34] observed that the inhibition of succinate dehydrogenase (SDH) in normoxia increased the accumulation of succinate, which consequently augmented the expression of HIF-1α in human embryonic kidney cells. They demonstrated that succinate inhibited the activity of PHD *in vitro* and thus might have stabilized HIF-1α protein. Consequently, Koivunen et al. [35] revealed that fumarate and succinate were potent inhibitors of all three PHDs and they also increased the expression of HIF-1α and its target gene *VEGF* in cultured cells. Pollard et al. [36] reported that the germline mutations of fumarate hydratase (*FH*) and *SDHB*, -*C*, and -*D* genes provoked the accumulation of fumarate and succinate into cultured cells and induced the over-expression of HIF-1α, which promoted the appearance of different types of cancers. These studies clearly indicated that fumarate and succinate were competitive inhibitors of Fe^2+^/2-oxoglutarate-dependent PHDs and thus stabilized HIF-1α expression in normoxia. Moreover, several studies have revealed that iron chelators are potent inducers of HIF-1α expression, such as 1,10-phenanthroline and flavonoid quercetin [37, 38]. Given that hypoxia tolerance can provide protection against ischemic and inflammatory diseases, many drug discovery studies have been launched to develop effective inhibitors to PHD enzymes [39].

In 1998, Zelzer et al. [40] demonstrated that insulin and IGF-1 treatments stimulated the formation of HIF-1α/HIF-1β complexes, which could bind to HRE sites in Hep-G2 and muscle L8 cells. These complexes also transactivated the expression of HIF-1α-dependent target genes. Subsequently, many studies confirmed that insulin and IGF-1 induced the HIF-1α signaling via the phosphatidylinositol 3-kinase/target of rapamycin (PI3K/mTOR) pathway [41-43]. Moreover, the expression of phosphatase and tensin homolog (PTEN), an antagonizing phosphatase for the PI3K/mTOR pathway, inhibited the accumulation of HIF-1α protein [41], whereas the loss of PTEN enhanced the PI3K/Akt-mediated HIF-1α signaling [44]. Recently, Seok et al. [45] demonstrated that HIF-1α stimulated the expression of miR-382, which targeted PTEN and thus could facilitate the PI3K/mTOR-mediated HIF-1α signaling, e.g. potentiate tumor angiogenesis. It seems that mTOR activates the HIF-1α signaling through increasing the transcription and translation of HIF-1α rather than inhibiting its degradation [43, 46]. There are observations that AMP-activated protein kinase (AMPK) can inhibit the activity of PI3K/mTOR/HIF-1α pathway [47]. In addition, mitogen-activated protein kinases (MAPK), especially p42/p44 MAPK, can phosphorylate HIF-1α protein and thus increase its transactivation capacity [48], whereas glycogen synthase kinase 3 (GSK-3) phosphorylates HIF-1α and consequently enhances its proteasomal degradation [49]. These observations indicate that the major protein kinase cascades can also control the HIF-1α signaling (Fig. 1).

Inflammatory milieu of tissues commonly involves hypoxia and thus directly triggers the PHD-dependent activation of HIF-1 signaling. However, a substantial literature indicates that cytokines and the NF-κB signaling can stimulate a pseudohypoxic HIF-1α expression through different mechanisms [50-52] (Fig. 1). Pro-inflammatory cytokines, especially interleukin-1β (IL-1β) and tumor necrosis factor-α (TNF-α), can increase the expression of HIF-1α protein, which subsequently augments the expression of target genes [53-55]. There seems to be an autocrine loop in inflammation since HIF-1α can bind to the HRE site at the promoter of *IL-1β* gene and stimulate its expression [55]. van Uden et al. [56] demonstrated that the NF-κB transcription factor induced the expression of HIF-1α protein, and moreover, they reported that HIF-1α expression promoted by TNF-α was mediated by the NF-κB signaling in normoxia. Sun et al. [52] revealed that TNF receptor-associated factor 6 (TRAF6), a major activator of NF-κB, could bind to HIF-1α protein and induce its K63-linked polyubiquitination, which stabilized HIF-1α protein against proteasomal degradation. On the other hand, an increased expression of HIF-1α can repress the NF-κB signaling and consequently suppress inflammatory responses [57]. Given that HIF-1α can stimulate the expression of PHD2/3 [25], there seems to be a negative feedback route to inhibit NF-κB system, enhanced by HIF-1α expression. The cross-talk between NF-κB and HIF-1α in inflammation might be organized in cell type and context-dependent manner. In addition to the NF-κB system, the JAK-STAT signaling, mediating cytokine and growth factor signaling, can also stimulate the expression of HIF-1α protein (Fig. 1). Jung et al. [58] demonstrated that signal transducer and activator of transcription 3 (STAT3) can bind to the HIF-1α protein and restrict the binding of pVHL ligase, thus stabilizing HIF-1α protein in normoxia. Moreover, there are observations that the TGF-β/Smad3 signaling can increase the expression of HIF-1α in normoxia by either inhibiting the expression of PHD2 [59] or activating the mTOR complex [60]. Sphingosine-1-phosphate (S1P) can also induce the expression of HIF-1α protein in normoxia, indicating that ceramide pathway might also control the HIF-1α signaling [61]. There may be different mechanisms since S1P can stimulate both the NF-κB and STAT3 signaling pathways, which are the activators of HIF-1α expression (see above). Nizet et al. [62] have reviewed the close interactions between hypoxia and innate immunity responses in the immune system and related diseases.

Aging and age-related degenerative diseases are associated with oxidative stress, probably partly linked to increased inflammation. Interestingly, there is abundant literature indicating that reactive oxygen species (ROS), such as hydrogen peroxide (H_2_O_2_) and nitric oxide (NO), can stabilize HIF-1α protein in normoxia [63, 64]. However, there is still debate whether ROS can directly stabilize HIF-1α in either normoxia or hypoxia [65, 66]. Mateo et al. [63] observed that increased NO production stabilized HIF-1α protein in mitochondria-dependent or - independent manner in cultured HEK-293 cells. Metzen et al. [67] reported that NO inhibit PHDs, and thus it could stabilize HIF-1α protein. This same mechanism could also inhibit other 2-OGDO enzymes, such as KDMs [68], probably controlling the iron redox status. It seems that ROS affect the HIF-1α signaling in a context-dependent manner and differently in cancer and ischemia [69].

The acetylation of HIF-1α protein is one of the post-translational modifications, which control both the stability of HIF-1α and its transcriptional activity. It is known that histone acetyltransferases, p300/CBP [70] and PCAF [71], can acetylate HIF-1α protein and thus increase its stability and expression level in cultured cells. On the other hand, Lim et al. [72] demonstrated that SIRT1, a class III protein deacetylase, could bind to and subsequently deacetylate HIF-1α protein, which blocked the recruitment of p300 acetyltransferase to target gene promoters, preventing the HIF-1α-dependent gene transcription. This repression was more important in normoxia, since the activity of NAD^+^-dependent SIRT1 is repressed in hypoxia, attributable to a decrease in the level of NAD^+^ in hypoxia. However, Chen et al. [73] demonstrated that HIF-1α increased the expression of SIRT1 in hypoxia, which might compensate the loss of SIRT1 activity. There are studies that some members of histone deacetylases (HDAC) of class IIA, such as HDAC5 [74], and HDAC7 [75], can increase the transcriptional activity of HIF-1α, although mechanisms are mostly elusive. Interestingly, Chen et al. [74] revealed that HDAC5 deacetylated heat shock protein 70 (HSP70) in the cytosol, which dissociated the complex between HSP70 and HIF-1α proteins and consequently increased the nuclear accumulation of HIF-1α, both under hypoxia and low glucose conditions. They also reported that the AMPK-induced phosphorylation of HDAC5 increased its transfer back to the cytosol and thus enhanced the capacity of HDAC5 to translocate HIF-1α proteins into the nuclei. HIF-1α is a client protein of HSP90, which interaction stabilizes HIF-1α protein against proteasomal degradation in normoxia [76]. Liu et al. [77] demonstrated that the receptor of activated protein kinase C (RACK1) competed with HSP90 for the binding to the same domain in HSP-1α protein. RACK1 is a multifunctional scaffold protein which stimulated the O_2_-independent proteasomal degradation of HIF-1α. Subsequently, Liu et al. [78] reported that calcineurin, a Ca^2+^-activated protein phosphatase, stimulated the expression of HIF-1α since it dephosphorylated RACK1, which blocked its dimerization and binding to HIF-1α protein. These observations indicate that disturbances in calcium homeostasis, observed in many age-related diseases, can stabilize HIF-1α protein in normoxia.

In conclusion, it seems that a plethora of stress-related signals in normoxia can stimulate the HIF-1α signaling and in that way enhance survival responses but also aggravated pathological processes associated with the HIF-1α signaling.

**Table 1:** HIF-1α-inducible histone lysine demethylases (KDMs)

KDM	Target	References
**KDM2B** FBXL10, JHDM1	**H3K4me3** & **H3K36me1,2**	[89]
**KDM3A** JMJD1A, JHDM2A	**H3K9me1,2**	[85-91, 93]
**KDM4B** JMJD2B	**H3K9me2,3** & **H3K36me2,3**	[85-87, 89, 91, 92]
**KDM4C** JMJD2C	**H3K9me2,3** & **H3K36me2,3**	[85, 86, 89]
**KDM5B** JARID1B	**H3K4me2,3**	[87, 89]
**KDM6B** JMJD3	**H3K27me2,3**	[89, 95]

The formerly used symbols of KDMs are marked below the official symbols.

## HIF-1α induces the expression of distinct histone lysine demethylases

DNA and histone methylation are the two major epigenetic mechanisms which regulate gene expression. The methylation status of histones is controlled by histone methyltransferases and histone demethylases [79]. The methylation of histone C-terminal lysines can either activate or repress gene expression, i.e. typical activating marks are histone 3 di- or trimethylated lysine 4 (H3K4me2,3) and H3K36me2,3, whereas H3K9me1,2 and H3K27me2,3 are common repressive sites (Table 1). There are six Jumonji C domain containing histone lysine demethylases (KDM2-7), which can remove both activating and repressing methyl groups in an enzyme specific manner [21, 80, 81]. For instance, the demethylases of KDM3 and KDM6 subgroups are the potent activators of gene expression erasing the methyl groups from the repressive H3K9 and H3K27 sites, respectively. The Jumonji-type of KDMs contain different protein-protein binding domains through which they can specifically interact with distinct chromatin proteins, such as histone deacetylases, transcription factors and other chromatin proteins [21, 80]. All Jumonji-type of histone demethylases are the members of 2-oxoglutarate-dependent dioxygenase family (2-OGDO); this means that they are dependent on the availability of oxygen and 2-oxoglutarate, a Krebs cycle intermediate, similarly to PHDs (see above). However, they are not as sensitive oxygen sensors as PHDs [82-84]. In 2008, Pollard et al. [85] screened the effects of hypoxia on the expression levels of several Jumonji-type of histone demethylases in many cultured cell lines. They observed that the expression of KDM3A and KDM4B mRNAs but not that of several other demethylases were significantly induced in U2OS, MCF7, HeLa, IMR32 and HL60 cells cultured in 0.5% O_2_ pressure. They also revealed that the promoters of *KDM3A* and *KDM4B* genes contained three putative HRE binding sites, which were not present in the promoters of the non-responsive demethylases. Moreover, they reported that HIF-1α proteins could directly bind to these sites at the promoters of *KDM3A* and *KDM4B* genes. Beyer et al. [86] also demonstrated that hypoxia stimulated the HIF-1α-dependent expression of KDM3A and KDM4B mRNAs and proteins in cultured cells (Table 1). Moreover, they observed that hypoxia did not affect the global levels of di- and trimethylated H3K9 indicating that KDM3A and KDM4B proteins have specific binding partners and distinct targets in the genome. Krieg et al. [87] reported that only a few of the hypoxia-inducible genes are dependent on the presence of KDM3A (53 out of 821) in RCC4 cells. This subset included e.g. adrenomedullin (*ADM*), heme oxygenase 1 (*HMOX1*), and *SERPINE1*. The KDM3A-induced increase in the expression of these genes was associated with a decrease in the level of repressive H3K9me2 marks at the target promoters. Many other studies have also reported that hypoxia stimulated the expression of *KDM3A* and *KDM4B* genes in different cellular contexts [88-92] (Table 1). Interestingly, Wellmann et al. [93] demonstrated that the normobaric hypoxia (8% O_2_) of rats robustly increased the expression of KDM3A in all tissues studied, e.g. brain, heart, and liver. Given that KDM3A and KDM4B are the major histone demethylases which remove the repressive H3K9 sites, their role as transcriptional cofactors seems to be important in the activation of HIF-1α signaling.

The di- and trimethylated H3K27 (H3K27me2,3) is another major repressive histone site in the control of gene transcription. Moreover, H3K27me3 marks are associated with the polycomb complexes, which can repress long genomic domains and also organize the chromosomal 3D structures [94]. KDM6A (UTX) and KDM6B (JMJD3) are the two major H3K27me2,3 demethylases [80, 81]. Recently, Lee et al. [95] demonstrated that the hypoxic conditions clearly increased the expression of KDM6B in a HIF-1α-dependent manner in NIH-3T3, 3T3-L1, and mouse embryonic fibroblasts (MEF) (Table 1). They also reported that the promoter and first intron of human *KDM6B* gene contained several HRE sites. Moreover, clioquinol, an iron chelator and pseudohypoxic inducer of HIF-1α expression, strongly induced the expression of KDM6B in human adipose-derived stem cells. In addition to HIF-1α, the expression of KDM6B can be stimulated by NF-κB, STAT3, and ATF4 signaling pathways [96]. A robust induction of KDM6B by HIF-1α suggests that KDM6B is an important hypoxia-inducible transcriptional cofactor, which demethylates repressive H3K27me2,3 sites, although its role in the modification of global chromatin landscape still needs to be clarified.

Xia et al. [89] used a genome-wide screening to identify the direct binding targets of HIF-1 transcription factor. They observed that *KDM4C* (*JMJD2C*) and *KDM5B* (*JARID1B*) also contained HRE sites for the binding of HIF-1 factors, in addition to the above mentioned demethylase genes (Table 1). The expression of these enzymes was significantly increased in HepG2 cells in the hypoxic conditions [89]. Moreover, Pollard et al. [85] and Beyer et al. [86] reported that the expression of *KDM4C* was modestly increased in cultured hypoxic cells. Accordingly, Krieg et al. [87] confirmed that *KDM5B* also was a hypoxia-inducible gene (Table 1). Moreover, the genome-wide screening by Xia et al. [89] revealed that the expression of KDM2D, KDM3B/3C, KDM5C, and KDM6A were up-regulated in a HIF-1α-dependent manner in Hep G2 cells. These observations have not been confirmed by other studies. It seems that hypoxia stimulates both activating and repressive epigenetic marks to enhance or inhibit the expression of distinct genes in hypoxic conditions although excessive activation can aggravate pathological processes.

## Oxygen deprivation inhibits the activity of histone lysine demethylases

Molecular oxygen is an obligatory co-substrate for the Jumonji-type histone demethylases [21] as well as for other 2-OGDO enzymes, e.g. PHD1-3 [18]. However, there is a great variation for the requirement of O2 tension in the catalytic mechanism of different 2-OGDO enzymes [68, 83, 86, 91, 97]. PHDs are clearly more sensitive hypoxic sensors than Jumonji-type histone demethylases. For instance, Beyer et al. [86] demonstrated that the 1% O2 tension did not affect the activity of transfected KDM3A and KDM4B, whereas the reduction of O2 pressure down to 0.2% compromised the activity of KDM3A and KDM4B. In contrast, it is known that a modest decrease in O2 level can stimulate a robust hypoxic response, e.g. in mouse kidney with a chronic treatment at 11% O2 pressure [98] or disturb coronary artery development in a HIF-1α-dependent manner at 15% O2 tension [99].

There is a complex situation in the control of Jumonji-type demethylases in the conditions where oxygen availability is reduced. Tausendschön et al. [91] demonstrated that the 1% O2 exposure robustly increased the expression of KDM3A and KDM4B in mouse macrophages, although simultaneously there was a slight increase in the methylation levels of H3K9me2,3 and H3K36me3, which might indicate a decrease in the activity of these demethylases. On the other hand, it is possible that the effects of hypoxia on the activity of Jumonji-type histone demethylases is not caused by the deficiency of O2 as a substrate but through the hypoxia-induced other responses, e.g. increased NO production (Fig. 1). Hickok et al. [68] demonstrated that the NO exposure inhibited the activity of KDM3A enzyme in vitro by forming a nitrosyl-iron complex in the catalytic domain of this 2-OGDO enzyme. They also reported that NO production in cultured cells increased the global level of H3K9me2 indicating an inhibition of KDM3A although other Jumonji-type of demethylases targeting H3K9me2 could also have been inhibited. Moreover, NO can inhibit PHDs, including in 2-OGDO enzymes, and thus stabilize HIF-1α protein [67]. This might also explain why the NO treatment increased the expression of KDM3A and some other Jumonji-type histone demethylases in cultured cells [68].

The hypoxia-induced alterations in the methylation status of H3K4, H3K9, H3K27, and H3K36, at either global or specific promoter level, are commonly explained as changes in the activities of specific KDMs. However, this is not the correct parameter since hypoxia also affects the expression of histone lysine methyltransferases. For instance, hypoxia stimulates the expression of G9a, an H3K9 site methyltransferase, in cultured cells [100]. Hypoxia can also increase the recruitment of histone methyltransferases to specific promoters, e.g. that of SuvH1 and SuvH2 (both are H3K9 methyltransferases) to the promoter of surfactant protein A (SP-A) gene [101]. Moreover, Set7, an H3K4 methyltransferase, can directly methylate HIF-1α and HIF-2α proteins and thus inhibit their transcriptional activity [102]. These examples emphasize the complexity to interpret the mechanisms behind the changes in histone methylation induced by hypoxic conditions.

## Hypoxia modifies histone methylation

Several studies have revealed that hypoxia provokes epigenetic changes in the chromatin landscape, which consequently affect the transcriptional profiles of tissues [28, 103-105]. However, hypoxia is a complex subject since results are dependent on many details in hypoxic treatments, e.g. the type of model (acute/chronic or constant/intermittent), oxygen level during exposure, and post-hypoxia timing, even transgenerational changes. Moreover, hypoxic training can induce hypoxic/ischemic tolerance [106], which involves an appearance of specific epigenetic signature [107]. Hypoxia tolerance is also associated with increased lifespan, e.g. in the case of naked mole rats [108]. Moreover, hypoxia is present in the pathogenesis of many age-related diseases, such as cardiovascular diseases and Alzheimer’s disease, which involve substantial epigenetic alterations in the chromatin landscape [109, 110]. Currently, it is not known how hypoxia generates chromatin changes, which can be either protective enhancing hypoxic tolerance or detrimental provoking pathological changes. However, it is clear that epigenetic alterations, such as the stimulation of distinct KDMs, are crucial mechanisms in the induction of HIF-1α-mediated hypoxic response [111] (Fig. 1).

Histone methylation in collaboration with DNA methylation has a key role in the control of chromatin structure and gene expression [112]. Histone demethylases control gene expression via different mechanisms, i.e. they can (i) enhance or repress the initiation of transcription by binding to the initiation complex and demethylating epigenetic histone marks, (ii) increase the rate of transcription elongation by binding to Pol II and removing methyl groups from the histones locating in gene bodies, (iii) recruit co-activators to the initiation complex in a demethylase-independent manner [80, 113]. There are many studies indicating that the hypoxia-inducible KDMs control gene expression through the histone demethylation of target gene promoters [87, 89, 92]. Fu et al. [92] demonstrated that the hypoxia-induced overexpression of KDM4B decreased the level of H3K9me3 at the promoters of target genes, whereas the depletion of KDM4B increased H3K9 methylation. Johnson et al. [114] reported that the promoters of hypoxia-activated genes (*VEGF* and *EGR1*) displayed a marked increase in the acetylation of H3K9 sites, while there was a significant loss in the levels of H3K9me2 and H3K27me2. In contrast, at the promoters of hypoxia-repressed genes (*AFP* and *ALP*), hypoxia caused a decrease in the acetylation of H3K9 sites, whereas the methylation of H3K9 and H3K27 increased. These examples underline the crucial role of hypoxia-inducible KDMs as the amplifiers of HIF-1α-mediated hypoxic stress responses. However, the induction of KDMs by hypoxia/pseudohypoxia might have other targets than HIF-1α-dependent genes, which could affect the global gene expression and even the maintenance of chromatin structure. For instance, the KDM4 family members are associated with carcinogenesis, i.e. they are highly expressed in many cancers and an experimental overexpression increased tumor growth and enhanced invasion [115, 116]. Toyokawa et al. [115] revealed that KDM4B promoted cell cycle progression by demethylating H3K9me3 sites at the promoter of cyclin-dependent kinase 6 (CDK6). Awwad and Ayoub [117] demonstrated that the overexpression of KDM4 genes increased genomic instability by disrupting the DNA mismatch repair system. It is known that the DNA mismatch repair pathway is impaired with aging and age-related pathologies [118]. Moreover, the overexpression of KDM6B can induce the expression of specific genes linked to the senescence-associated secretory phenotype (SASP) and consequently provoke cellular senescence including blebbing of nuclei in glioma cells [119]. The nuclear blebbing indicates profound disturbances in the polycomb-driven 3D maintenance of nuclear structure, which we will discuss later.

Severe chronic hypoxia, as commonly used in experimental models, induces a reduction in gene transcription and protein synthesis, which consequently impair the appearance of HIF-1α-dependent responses [114, 120]. Most probably this also affects histone methylation during hypoxia since some processes are dependent on protein synthesis, e.g. hypoxia-inducible KDMs, whereas others are independent. This could explain why the results on global histone methylation during hypoxia are inconclusive. Many studies have detected a hypermethylation e.g. in H3K9me2,3, and H3K36me3 levels [83, 84, 105]. This is in line with the repression of transcription observed during hypoxia. As discussed earlier, hypoxia also stimulates the expression of G9a, which could induce the hypermethylation of H3K9 sites [100]. However, it is not known whether this is a counteraction to the induction of KDM3A and KDM4B. Interestingly, many studies have indicated that hypoxia also induces global DNA hypermethylation in several model systems [121-123]. This effect seems to be linked to the induction of DNA methyltransferases 1 (DNMT 1) and 3b (DNMT3b) in hypoxia. Currently, it is recognized that histone methylation can control DNA methylation and *vice versa* [112]. DNA methylation is a more permanent epigenetic mark than histone methylation and thus disturbances in histone methylation status might trigger profound changes in chromatin landscape, e.g. with aging and in associated diseases.

## Hypoxia-inducible histone demethylases control chromatin landscape and function

Jumonji histone demethylases have many important functions in addition to the gene-specific activation or repression of HIF-1α-dependent transcription. KDM proteins contain specific binding domains, which can mediate interactions between KDMs and several chromatin proteins as well as some DNA loci. This means that hypoxia and pseudohypoxia affect not only gene expression but also chromatin structures, e.g. the maintenance of heterochromatin and polycomb complexes which can silence large genome sequences and preserve chromosome configuration. Both the constitutive and facultative heterochromatin involve DNA regions with increased histone methylation of H3K9, H3K27, and H3K36 sites, which are assembled by heterochromatin protein 1 (HP1) and histone methyltransferases, such as those in polycomb complexes [124-126]. Heterochromatin is a platform for the binding of chromatin effector proteins participating in e.g. gene silencing and the formation of 3D higher-order chromosome foldings [94]. However, heterochromatin regions, especially facultative segments, are dynamic structures since HP1 proteins can recruit Jumonji histone demethylases, which activate the transcription in heterochromatin repeats [127-129]. In particular, the hypoxia-inducible H3K9 and H3K27 demethylases have a key role in the dissociation of heterochromatin segments. Transient hypoxia typically causes a short-term induction of HIF-1α signaling and changes in gene expression, whereas chronic exposure to hypoxia can have detrimental effects on heterochromatin maintenance and DNA integrity (Fig. 1).

There is substantial evidence that the overexpression of KDM3, KDM4, and KDM6 demethylases decreases the methylation level of repressive H3K9 and H3K27 marks (Table 1) and reduces the amount of heterochromatin, a condition which is commonly associated with e.g. cancers [116, 130, 131]. On the other hand, an increased expression of KDM2 and KDM5 down-regulate the methylation of H3K4 sites and thus enhances the maintenance of heterochromatin segments and genome stability [132]. In addition to histone demethylase-dependent functions, many KDMs also have enzyme-independent, linker protein properties via their different protein-protein binding domains. Abe et al. [133] demonstrated that KDM3A has a key role in the β-adrenergic-induced gene expression by interacting with the SWI/SNF nucleosome remodelling complex. Protein kinase A (PKA) phosphorylated KDM3A at S265, which subsequently triggered its interaction with the SWI/SNF complex. Consequently, this larger complex was located to the target gene promoters with peroxisome proliferator-activated receptor γ (PPARγ) transcription factor. Currently, it is not known whether KDM3A is involved in the other activities of the SWI/SNF complex, e.g. those of DAF-16/FOXO-mediated stress resistance and increased longevity [134]. Mimura et al. [135] reported that KDM3A interacted with HIF-1α at the promoter of *GLUT3* gene in hypoxic vascular endothelial cells increasing the expression of GLUT3 and subsequently enhancing glycolysis. They also revealed that the binding of KDM3A to HIF-1α induced changes in the chromatin conformation at the *SLC2A* (*GLUT3*) locus under hypoxia. This indicates that KDM3A affects chromatin structures to facilitate transcription in hypoxia.

There is an interesting diversity how the members of KDM4 subfamily respond to hypoxia, i.e. the expression of KDM4B and KDM4C increases but that of KDM4A and KDM4D is unaffected [85, 86, 89]. All KDM4 enzymes are highly expressed in many cancers, most likely they have different functions in carcinogenesis [116]. KDM4A has an important role in the regulation of heterochromatic loci in the collaboration with HP1α. Some studies indicate that HP1α can target and consequently stimulate the activity of KDM4A [128], although some experiments suggest that HP1α can antagonize the function of KDM4A [136]. KDM4A can also induce a site-specific increase in gene copy number, a typical phenomenon in cancers [137]. Moreover, KDM4 members are involved in the repair of DNA damages [138-140]. Zheng et al. [138] demonstrated that tumor suppressor p53 protein promoted heterochromatin DNA repair by inducing the expression of hypoxia-inducible KDM4B, which enhanced the relaxation of constitutive heterochromatin and thus improved DNA repair. In addition, Young et al. [140] reported that γ-irradiation recruited KDM4B to DNA damages in a PARP-1 -dependent manner and that the overexpression of KDM4B enhanced the repair of double-strand breaks (DSB). However, Awwad and Ayoub [117] observed that the overexpression of KDM4A-C, especially that of KDM4C, could disrupt the integrity of the DNA mismatch repair system, subsequently impairing the maintenance of genomic stability. These studies indicate that the members of KDM4 subfamily have a crucial role in the DNA repair systems, although the responses seem to be enzyme-specific and appear in a context-dependent manner.

The CpG islands are important DNA sequences in gene promoters controlling gene expression [141]. KDM2A and KDM2B proteins contain a domain called ZF-CxxC, which mediates the binding of these demethylases to non-methylated CpG islands (CGI) [21]. In 2012, Farcas et al. [142] demonstrated that both KDM2A and KDM2B proteins interacted with non-methylated CGIs, and remarkably, those sites enriched with KDM2B also contained protein components of polycomb repressive complex 1 (PRC1). Farcas et al. [142] and Wu et al. [143] revealed that KDM2B interacted with the RING1B component of PRC1 complex, which catalyzed the lysine 119 monoubiquitylation of histone H2A (H2AK119ub1). The ubiquitylated H2AK recruited the PRC2 complex to non-methylated CGI sites, and consequently the EZH2 component, a H3K27 methyltransferase, trimethylated H3K27 sites repressing the target gene expression [144, 145]. Moreover, Tzatsos et al. [146] demonstrated that the overexpression of KDM2B repressed the expression of two microRNAs, *let-7* and *miR-101*, which are silencers of EZH2 methyltransferase, and thus KDM2B increased the capacity of PRC2 complex to enhance H3K27 methylation, e.g. in the INK4 box preventing cellular senescence (see below). Frescas et al. [147] observed that KDM2B was localized in the nucleolus, where it blocked the transcription of RNA genes. This indicates that KDM2B has a significant role in the regulation of repressive polycomb system and thus it can control, e.g. developmental processes, cancer formation, and cellular senescence [148, 149]. For instance, KDM2B promotes the generation of induced pluripotent stem cells (iPSC) [150].

The four members of human KDM5 subfamily of Jumonji demethylases (KDM5A-D) demethylate H3K4me2,3 and thus they repress gene expression [21, 81]. The subfamily has also been called JARID1A-D, since they contain an AT-rich interaction domain (ARID), which mediates the DNA binding of these enzymes. They also comprise the PHD (plant homeobox domain) and zinc finger C5HC2 domains. It is known that KDM5B enzyme is included in many chromatin modifying complexes, such as the nucleosome remodelling and deacetylase complex (NuRD) [151, 152], PRC2 complex [153], and SWI/SNF complex [154]. KDM5B is a potent inhibitor of gene expression not only through the demethylase domain but it also interacts with histone deacetylases (HDAC), those of class I and IIa HDACs [152, 155]. On the other hand, poly (ADP-ribose) polymerase-1 (PARP-1) binds to KDM5B protein and inhibits its activity by poly(ADP-ribosyl)ation [156]. Through this activity, PARP-1 can maintain open chromatin architecture and activate target gene expression. KDM5B can also be SUMOylated, which affects its recruitment to target genes but does not inhibit its demethylase activity [157]. Hendriks et al. [158] demonstrated that DNA damages induced the SUMOylation of KDM5B and KDM5C proteins. Subsequently, the SUMOylated KDM5B protein was ubiquitylated and degraded by proteasomes. Instead, SUMOylated KDM5C was recruited to the chromatin complexes, where it demethylated H3K4me2,3 sites inhibiting gene expression. KDM5B has an important role in tissue differentiation during development, since in association with polycomb complexes it silences the lineage-inappropriate genes, e.g. in neural differentiation [159, 160].

KDM6A (UTX) and KDM6B (JMJD3) demethylate repressive H3K27me2,3 marks in the transcription of genes as well as they antagonize the polycomb-induced gene silencing processes and enhance the displacement of polycomb complexes from the specific gene loci, e.g. from the INK4 box (see below). In view of these functions, KDM6 demethylases have a fundamental role in programming of chromatin structures during developmental processes. Saha et al. [161] demonstrated that the first mammalian cell lineage commitments during blastocyst formation are regulated by KDM6B. There is substantial evidence that KDM6B regulates e.g. the neural commitment and neurogenesis [162], endodermal [163], mesodermal and cardiovascular differentiation [164]. Dahle et al. [165] demonstrated that the Nodal-Smad2/3 signaling recruited KDM6B to *Brachyury* locus to displace repressive polycomb complexes in embryonal stem cells to trigger the formation of mesoderm and endoderm. T-box transcription factors recruit KDM6B proteins to specific gene loci to switch on cell-type specific gene expression patterns [166]. The T-bet protein induced commitment of CD4^+^ T cells into different Th subtypes [167] and the activation of *Eomes* gene locus in endodermal differentiation [163] are some examples of the cooperation between KDM6B and T-box factors. Given that KDM6B can regulate the developmental programs, it is interesting that KDM6B can also activate the INK4 box (see below), containing tumor suppressor genes, which induce cellular senescence [168]. Recent studies have revealed that cellular senescence has fundamental role in the aging process and age-related diseases. We will discuss the function of KDM6B in the regulation of INK4B box in the next section.

## Does excessive HIF-1α stimulation disturb chromatin landscape with aging and associated diseases?

Currently, it seems that HIF-1α signaling can have positive and negative effects on the regulation of longevity [169]. There are some dramatic examples that the living in hypoxic conditions is associated with a significant increase in the lifespan of animals, e.g. subterranean blind mole rats can live for 30 years [108] and ocean quahog *Arctica islandica* for hundreds of years [170]. Shams et al. [171] demonstrated that the expression of HIF-1α was not only higher in normoxic kidney of *Spalax* compared to *Rattus norvegicus* but the induction of HIF-1α expression was especially enhanced in hypoxia, being 6-fold higher in *Spalax* than *Rattus norvegicus*. However, it is not known whether increased hypoxic stress tolerance is related to longer lifespan. There are many reports indicating that the stimulation of HIF-1α expression in hypoxia is declined with aging [172-174]. Interestingly, Ndubuizu et al. [173] revealed that the age-dependent decline in hypoxia-inducible expression of HIF-1α was correlated with an increased expression of PHD1 in rat brain. Rohrbach et al. [175] reported that the expression of PHD3 was significantly increased in rat heart, liver, and skeletal muscle with aging. An increased expression of PHDs can be a negative feedback response to increased HIF-1α expression, since some studies have demonstrated that HIF-1α induces the transcription of PHDs and thus protects against the chronic stimulation of HIF-1α [25, 176].

Hypoxia-dependent regulation is not the only mechanism, which can stimulate the HIF-1α signaling (Fig. 1). There are a number of cellular signaling pathways, which can induce the transcription of HIF-1α factor, stabilize its expression, or enhance its transactivation capacity (see above). Interestingly, many of these pathways are associated with the ageing process and age-related diseases. For instance, there is an abundant literature indicating that excessive oxidative stress can be a causative factor in the aging process and many age-related diseases [177]. Oxidative stress is commonly linked to a chronic, low-grade inflammation and the activation of NF-κB signaling [178, 179], also an inducer of HIF-1α expression. The ceramide and TGF-β signaling pathways are involved in the pathogenesis of many age-related diseases, such as atherosclerosis [180, 181]. The insulin/PI3K/mTor signaling pathway is a well-known regulator of longevity through the species [182]. Moreover, dysfunctions in mitochondrial metabolism can affect both the aging process and age-related diseases [183]. All the above-mentioned mechanisms can increase the level of HIF-1α and thus stimulate the expression of hypoxia-inducible KDMs in normoxia (see above). There is substantial evidence that the HIF-1α signaling has a protective function in acute insults, e.g. in ischemic conditions [184], whereas the effect seems to be harmful in chronic diseases, such as age-related macular degeneration (AMD) [185], cancer progression [186], chronic kidney disease [187], cardiomyopathies [188], and adipose tissue fibrosis and inflammation [189]. Most of these chronic responses are caused by damaging effects of excessive angiogenesis and fibrosis induced by HIF-1α. It appears that there is no negative feedback loop in the PHD-independent control of HIF-1α signaling (Fig. 1). This may cause detrimental effects, which are linked to epigenetic changes, e.g. in fibrotic lesions of myocardium and atherosclerotic arteries [188, 190, 191]. Recent epigenetic studies have clearly shown that the aging process is associated with significant changes in the chromatin landscape [192-194]. Genome-wide DNA methylation screenings have revealed a so-called epigenetic drift, which shows a decline in global DNA methylation although distinct DNA regions, e.g. CpG islands, display an increase in DNA methylation with aging. Moreover, it was observed that the progeroid mutations of Hutchinson-Gilford and Werner syndromes abundantly promoted the age-related changes in DNA methylation and chromatin structure, e.g. decrease in the amounts of H3K9me3 and constitutive heterochromatin [195-197]. Loss of heterochromatin also is a typical hallmark in cellular senescence and aging process [198-200], which impairs the maintenance of DNA integrity with aging. Larson et al. [199] reported that enhanced heterochromatin formation promoted longevity in *Drosophila*. Concurrently, the aging process is linked to global decrease in the levels of H3K9me3 and H3K27me3, repressive epigenetic marks [201]. Given that gene silencing by polycomb complexes and heterochromatin loci are dependent on trimethylation of H3K9 and H3K27 sites, it implies that the activation of KDM4 and KDM6 demethylases might induce changes in chromatin landscape with aging. Fodor et al. [130] reported that KDM4B antagonized the generation of H3K9me3 at the pericentric heterochromatin. Recently, many studies have revealed that there are remarkable epigenetic changes in atherosclerotic vessels, e.g. reduced levels of H3K9me2,3 and H3K27me2,3 [190, 191]. Epigenetic alterations have also been reported in cardiovascular diseases [202] as well as in obesity and metabolic syndrome [203]. Angiogenesis, critically involved in cancer and AMD progression, can be augmented by the stimulation of KDM3A [204], KDM5B [205], and KDM6B [206], the expression of which is highly enhanced by HIF-1α (Table 1).

Cellular senescence, i.e. an irreversible cell growth arrest, is associated with remarkable global alterations in chromatin landscape [207, 208] along with changes in cellular morphology, metabolism, and function [209]. Several studies have demonstrated that the presence of senescent cells in tissues increases with aging [210, 211] as well as in chronic age-related degenerative diseases [212, 213]. Senescent cells secrete many inflammatory mediators, growth factors and proteases, and thus they can promote the aging process and age-related diseases [209, 214]. The INK4 box, containing *INK4a*, *INK4b*, and *ARF* genes, is a crucial tumor suppressor locus and an inducer of cellular senescence [168, 215]. INK4a (p16) and INK4b (p15) are cyclin D-dependent kinase inhibitors, while ARF (p19) is an activator of p53-dependent functions. It is known that the expression of these factors controls not only proliferation but also cellular senescence. A substantial literature indicates that the function of INK4 box is under a tight epigenetic regulation [216, 217]. The INK4 box is repressed in proliferating cells through the local transcription of ANRIL, a non-coding RNA, which recruits the PRC2 complex to the INK4 box. Subsequently, the EZH2 methyltransferase of PRC2 trimethylates H3K27 sites and silences the transcription in the INK4 locus. KDM6B have a key role in the activation of transcription in the INK4 box since it demethylates H3K27me3 sites and releases polycomb complexes from the locus [218-220]. Different types of stresses can activate the transcription in the INK4 box and trigger cellular senescence through the demethylation of H3K27me3 sites and thus displacing polycomb complexes. Given that most of the pseudohypoxic inducers of HIF-1α are associated with cellular stress, it is conceivable that an increased expression of KDM6B can provoke the transcription in the INK4 locus. Interestingly, the *ANRIL* gene is a genetic susceptible locus for many age-related diseases [221].

It is known that the retinoblastoma (RB) tumor suppressor pathway has a major role in the onset of cellular senescence [209]. There are observations that KDM5B can trigger cellular senescence in association with the RB pathway [222, 223]. RB protein recruits KDM5B to the promoters of E2F target genes, many of which are cell-cycle regulators, silencing their expression and subsequently inducing senescence. Narita et al. [224] observed that the formation of senescence-associated heterochromatic foci (SAHF) was also triggered by the RB-dependent inhibition of E2F genes, probably through the KDM5B-mediated H3K4 demethylation. It appears that KDM6B protein activates the expression of tumor suppressors in the INK4 box, whereas KDM5B proteins directly inhibit the transcription of E2F-dependent cell-cycle factors. However, the role of HIF-1α in the generation of cellular senescence seems to be more complicated. There are observations that acute hypoxia can stimulate cell-cycle arrest but does not provoke cellular senescence in all contexts, even it can suppress the oncogene-induced senescence [225, 226]. There are studies indicating that mTOR activation is required to trigger the conversation of arrested cells to senescent ones (geroconversion) [227]. The nuclear translocation of p53, mediated by KDM6B, can also enhance the geroconversion through the expression of p21/CIP1, a cyclin-dependent kinase inhibitor [228]. On the other hand, hypoxia and HIF-1α stimulate the expression of KDM2B and KDM5B, which can repress gene expression through the demethylation of activating H3K4me2,3 marks in the INK4 box and thus enhance proliferation [148] (see above). It seems that HIF-1α can control cellular fate in adult animals, either stimulating proliferation or triggering cellular senescence, by regulating the expression of different KDMs in a context dependent manner.

The HIF-1α signaling has an important role in the innate immunity responses, e.g. in inflammation, macrophage polarization, and T cell activation [229]. It is known that KDM6B has important functions in immune host defence responses. We have recently reviewed the role of KDM6B in the regulation of inflammatory responses associated with the aging process [96]. Given that cellular senescence is linked with inflammatory phenotype [209], it is interesting that KDM6B is a potent enhancer of inflammatory genes, especially in association with NF-κB signaling [230, 231]. Remarkably, the NF-κB pathway is an important inducer of cellular senescence-associated secretory phenotype (SASP) [232]. Moreover, there are studies indicating that KDM6B is an important activator of TGF-β signaling pathways by binding to Smad3 factor [165]. Subsequently, Smad3 can cooperate with NF-κB signaling [233]. The TGFβ-Smad3 signaling controls fibrosis and inflammation, e.g. in chronic kidney diseases [234]. Perrigue et al. [119] observed that the overexpression of KDM6B induced the expression of genes related to the SASP. Zhao et al. [235] observed that the level of KDM6B protein was significantly increased in senescent cells and it promoted the formation of heterochromatic SAHF foci. Cells expressing the properties of SASP accumulate during ageing and age-related diseases, although their role in the tissue ageing process and pathology needs to be clarified [214].

Hypoxia is an important inducer of fibrogenesis, or fibrosis, in many tissues and especially, it is linked to the pathogenesis of many age-related diseases [189, 236, 237]. Pathological fibrosis involves excessive deposition of fibrous connective tissue into healthy or injured organs, e.g. myocardium, lungs, kidney, and adipose tissue. There seems to be two mechanisms, (i) the conversion of fibroblasts or other stromal cells to myofibroblasts and (ii) the transition of epithelial or endothelial cells to mesenchymal cells, i.e. the epithelial-mesenchymal transition (EMT). EMT is also a critical mechanism in embryonal morphogenesis and cancer metastasis [238]. The HIF-1α and TGF-β signaling pathways are the major inducers of EMT, although signaling networks also contain other factors in a context dependent manner [239-241]. Many studies have indicated that epigenetic factors control the hypoxia-induced EMT, as reviewed by Stadler and Allis [242] and Wu et al. [241]. Ramadoss et al. [243] demonstrated that KDM6B promotes the TGF-β-induced EMT by enhancing *Snai1* expression in mammary epithelial cells, whereas its knockdown prevented EMT. Dahle et al. [165] revealed that the TGF-β/Nodal signaling activated the *Brachyury* locus by recruiting Smad2/3 and KDM6B to its promoter in embryonal stem cells. It is known that the induction of Brachyury expression promotes interstitial fibrosis in renal tubuli [244]. The Brachyury protein is included in the T-box transcription factors. Given that KDM6B cooperates with T-box factors in cell lineage commitment (see above), it is obvious that KDM6B also drives EMT fibrosis in adult tissues as soon as the HIF-1α and TGF-β signaling pathways are excessively activated in pathological conditions. The stimulation of KDM6B and T-box factors reorganize the chromatin structures and consequently induce either cancerous growth or fibrogenesis, which is a common age-related deleterious response in many tissues.

## Conclusions

The HIF-1α signaling is a principal survival mechanism in acute hypoxic insults, where hypoxia-inducible KDMs are confined to support the transcription of genes comprising hypoxia response. However, it can be converted to detrimental response in some host defence condition, e.g. in chronic hypoxia and long-term pathological stresses, as soon as the activation of KDMs disturbs the maintenance of chromatin structures, e.g. heterochromatin repressed DNA segments and 3D folding of chromosomes, or stimulates embryonal processes, such as the EMT fibrosis. It seems that the HIF-1α-inducible KDMs have a crucial role in the activation of genes in the INK4 box as well as the RB-stimulated tumor suppressor genes, which both provoke a cell fate called cellular senescence. Senescent cells are deleterious for neighbouring cells since they secrete inflammatory mediators and thus they can activate signaling pathways known to stabilize the HIF-1α signaling. Given that cellular senescence is an irreversible alteration, it provides an inflammatory milieu which activates the HIF-1α-inducible KDMs and thus jeopardizes the nearby cells. This vicious cycle is increasingly present in the aging process and especially in the age-related diseases.

## References

[1] Kaelin WG Jr, Ratcliffe PJ (2008). Oxygen sensing by metazoans: the central role of the HIF hydroxylase pathway. Mol Cell, 30:393-402. 18498744 10.1016/j.molcel.2008.04.009

[2] Taylor CT, McElwain JC (2010). Ancient atmospheres and the evolution of oxygen sensing via the hypoxia-inducible factor in metazoans. Physiology (Bethesda), 25:272-279. 20940432 10.1152/physiol.00029.2010

[3] Rytkönen KT, Williams TA, Renshaw GM, Primmer CR, Nikinmaa M (2011). Molecular evolution of the metazoan PHD-HIF oxygen-sensing system. Mol Biol Evol, 28:1913-1926. 21228399 10.1093/molbev/msr012

[4] Semenza GL (2012). Hypoxia-inducible factors in physiology and medicine. Cell, 148:399-408. 22304911 10.1016/j.cell.2012.01.021 PMC3437543

[5] Ratcliffe PJ (2013). Oxygen sensing and hypoxia signalling pathways in animals: the implications of physiology for cancer. J Physiol, 591:2027-2042. 23401619 10.1113/jphysiol.2013.251470 PMC3634517

[6] Bigham AW, Lee FS (2014). Human high-altitude adaptation: forward genetics meets the HIF pathway. Genes Dev, 28:2189-2204. 25319824 10.1101/gad.250167.114 PMC4201282

[7] Larson J, Drew KL, Folkow LP, Milton SL, Park TJ (2014). No oxygen? No problem! Intrinsic brain tolerance to hypoxia in vertebrates. J Exp Biol, 217:1024-1039. 24671961 10.1242/jeb.085381 PMC3966918

[8] Semenza GL, Wang GL (1992). A nuclear factor induced by hypoxia via de novo protein synthesis binds to the human erythropoietin gene enhancer at a site required for transcriptional activation. Mol Cell Biol, 12:5447-5454. 1448077 10.1128/mcb.12.12.5447-5454.1992 PMC360482

[9] Wang GL, Semenza GL (1993). General involvement of hypoxia-inducible factor 1 in transcriptional response to hypoxia. Proc Natl Acad Sci U S A, 90:4304-4308. 8387214 10.1073/pnas.90.9.4304 PMC46495

[10] Wang GL, Semenza GL (1995). Purification and characterization of hypoxia-inducible factor 1. J Biol Chem, 270:1230-1237. 7836384 10.1074/jbc.270.3.1230

[11] Wang GL, Jiang BH, Rue EA, Semenza GL (1995). Hypoxia-inducible factor 1 is a basic-helix-loop-helix-PAS heterodimer regulated by cellular O2 tension. Proc Natl Acad Sci U S A, 92:5510-5514. 7539918 10.1073/pnas.92.12.5510 PMC41725

[12] Pugh CW, Tan CC, Jones RW, Ratcliffe PJ (1991). Functional analysis of an oxygen-regulated transcriptional enhancer lying 3′ to the mouse erythropoietin gene. Proc Natl Acad Sci U S A, 88:10553-10557. 1961720 10.1073/pnas.88.23.10553 PMC52967

[13] Firth JD, Ebert BL, Pugh CW, Ratcliffe PJ (1994). Oxygen-regulated control elements in the phosphoglycerate kinase 1 and lactate dehydrogenase A genes: similarities with the erythropoietin 3′ enhancer. Proc Natl Acad Sci U S A, 91:6496-6500. 8022811 10.1073/pnas.91.14.6496 PMC44229

[14] Ratcliffe PJ, O'Rourke JF, Maxwell PH, Pugh CW (1998). Oxygen sensing, hypoxia-inducible factor-1 and the regulation of mammalian gene expression. J Exp Biol, 201:1153-1162. 9510527 10.1242/jeb.201.8.1153

[15] Bruick RK, McKnight SL (2001). A conserved family of prolyl-4-hydroxylases that modify HIF. Science, 294:1337-1340. 11598268 10.1126/science.1066373

[16] Epstein AC, Gleadle JM, McNeill LA, Hewitson KS, O'Rourke J, Mole DR, et al (2001). C. *elegans* EGL-9 and mammalian homologs define a family of dioxygenases that regulate HIF by prolyl hydroxylation. Cell, 107:43-54. 11595184 10.1016/s0092-8674(01)00507-4

[17] Jaakkola P, Mole DR, Tian YM, Wilson MI, Gielbert J, Gaskell SJ, et al (2001). Targeting of HIF-α to the von Hippel-Lindau ubiquitylation complex by O2-regulated prolyl hydroxylation. Science, 292:468-472. 11292861 10.1126/science.1059796

[18] Schofield CJ, Ratcliffe PJ (2004). Oxygen sensing by HIF hydroxylases. Nat Rev Mol Cell Biol, 5:343-354. 15122348 10.1038/nrm1366

[19] Hausinger RP (2004). FeII/α-ketoglutarate-dependent hydroxylases and related enzymes. Crit Rev Biochem Mol Biol, 39:21-68. 15121720 10.1080/10409230490440541

[20] McDonough MA, Loenarz C, Chowdhury R, Clifton IJ, Schofield CJ (2010). Structural studies on human 2-oxoglutarate dependent oxygenases. Curr Opin Struct Biol, 20:659-672. 20888218 10.1016/j.sbi.2010.08.006

[21] Klose RJ, Kallin EM, Zhang Y (2006). JmjC-domain-containing proteins and histone demethylation. Nat Rev Genet, 7:715-727. 16983801 10.1038/nrg1945

[22] Pastor WA, Aravind L, Rao A (2013). TETonic shift: biological roles of TET proteins in DNA demethylation and transcription. Nat Rev Mol Cell Biol, 14:341-356. 23698584 10.1038/nrm3589 PMC3804139

[23] Salminen A, Kauppinen A, Kaarniranta K (2015). 2-Oxoglutarate-dependent dioxygenases are sensors of energy metabolism, oxygen availability, and iron homeostasis: Potential role in the regulation of aging process. Cell Mol Life Sci, doi:10.1007/s00018-015-1978-z. PMC11114064 26118662

[24] Makino Y, Cao R, Svensson K, Bertilsson G, Asman M, Tanaka H, et al (2001). Inhibitory PAS domain protein is a negative regulator of hypoxia-inducible gene expression. Nature, 414:550-554. 11734856 10.1038/35107085

[25] Marxsen JH, Stengel P, Doege K, Heikkinen P, Jokilehto T, Wagner T, et al (2004). Hypoxia-inducible factor-1 (HIF-1) promotes its degradation by induction of HIF-α-prolyl-4 hydroxylases. Biochem J, 381:761-767. 15104534 10.1042/BJ20040620 PMC1133886

[26] Loboda A, Jozkowicz A, Dulak J (2012). HIF-1 versus HIF-2 - is one more important than the other? Vascul Pharmacol, 56:245-251. 22366374 10.1016/j.vph.2012.02.006

[27] Schödel J, Mole DR, Ratcliffe PJ (2013). Pan-genomic binding of hypoxia-inducible transcription factors. Biol Chem, 394:507-517. 23324384 10.1515/hsz-2012-0351

[28] Rocha S (2007). Gene regulation under low oxygen: holding your breath for transcription. Trends Biochem Sci, 32:389-397. 17624786 10.1016/j.tibs.2007.06.005

[29] Mole DR, Blancher C, Copley RR, Pollard PJ, Gleadle JM, Ragoussis J, et al (2009). Genome-wide association of hypoxia-inducible factor (HIF)-1α and HIF-2α DNA binding with expression profiling of hypoxia-inducible transcripts. J Biol Chem, 284:16767-16775. 19386601 10.1074/jbc.M901790200 PMC2719312

[30] Majmundar AJ, Wong WJ, Simon MC (2010). Hypoxia-inducible factors and the response to hypoxic stress. Mol Cell, 40:294-309. 20965423 10.1016/j.molcel.2010.09.022 PMC3143508

[31] Greer SN, Metcalf JL, Wang Y, Ohh M (2012). The updated biology of hypoxia-inducible factor. EMBO J, 31:2448-2460. 22562152 10.1038/emboj.2012.125 PMC3365421

[32] Nallamshetty S, Chan SY, Loscalzo J (2013). Hypoxia: a master regulator of microRNA biogenesis and activity. Free Radic Biol Med, 64:20-30. 23712003 10.1016/j.freeradbiomed.2013.05.022 PMC3762925

[33] Chan SY, Loscalzo J (2010). MicroRNA-210: a unique and pleiotropic hypoxamir. Cell Cycle, 9:1072-1083. 20237418 10.4161/cc.9.6.11006 PMC2912143

[34] Selak MA, Armour SM, MacKenzie ED, Boulahbel H, Watson DG, Mansfield KD, et al (2005). Succinate links TCA cycle dysfunction to oncogenesis by inhibiting HIF-α prolyl hydroxylase. Cancer Cell, 7:77-85. 15652751 10.1016/j.ccr.2004.11.022

[35] Koivunen P, Hirsilä M, Remes AM, Hassinen IE, Kivirikko KI, Myllyharju J (2007). Inhibition of hypoxia-inducible factor (HIF) hydroxylases by citric acid cycle intermediates: possible links between cell metabolism and stabilization of HIF. J Biol Chem, 282:4524-4532. 17182618 10.1074/jbc.M610415200

[36] Pollard PJ, Briere JJ, Alam NA, Barwell J, Barclay E, Wortham NC, et al (2005). Accumulation of Krebs cycle intermediates and over-expression of HIF1α in tumours which result from germline FH and SDH mutations. Hum Mol Genet, 14:2231-2239. 15987702 10.1093/hmg/ddi227

[37] Wilson WJ, Poellinger L (2002). The dietary flavonoid quercetin modulates HIF-1α activity in endothelial cells. Biochem Biophys Res Commun, 293:446-450. 12054621 10.1016/S0006-291X(02)00244-9

[38] Park SS, Bae I, Lee YJ (2008). Flavonoids-induced accumulation of hypoxia-inducible factor (HIF)-1α/2α is mediated through chelation of iron. J Cell Biochem, 103:1989-1998. 17973296 10.1002/jcb.21588

[39] Fraisl P, Aragones J, Carmeliet P (2009). Inhibition of oxygen sensors as a therapeutic strategy for ischaemic and inflammatory disease. Nat Rev Drug Discov, 8:139-152. 19165233 10.1038/nrd2761

[40] Zelzer E, Levy Y, Kahana C, Shilo BZ, Rubinstein M, Cohen B (1998). Insulin induces transcription of target genes through the hypoxia-inducible factor HIF-1α/ARNT. EMBO J, 17:5085-5094. 9724644 10.1093/emboj/17.17.5085 PMC1170836

[41] Jiang BH, Jiang G, Zheng JZ, Lu Z, Hunter T, Vogt PK (2001). Phosphatidylinositol 3-kinase signaling controls levels of hypoxia-inducible factor 1. Cell Growth Differ, 12:363-369. 11457733

[42] Stiehl DP, Jelkmann W, Wenger RH, Hellwig-Bürgel T (2002). Normoxic induction of the hypoxia-inducible factor 1α by insulin and interleukin-1β involves the phosphatidylinositol 3-kinase pathway. FEBS Lett, 512:157-162. 11852072 10.1016/s0014-5793(02)02247-0

[43] Treins C, Giorgetti-Peraldi S, Murdaca J, Semenza GL, Van Obberghen E (2002). Insulin stimulates hypoxia-inducible factor 1 through a phosphatidylinositol 3-kinase/target of rapamycin-dependent signaling pathway. J Biol Chem, 277:27975-27981. 12032158 10.1074/jbc.M204152200

[44] Zundel W, Schindler C, Haas-Kogan D, Koong A, Kaper F, Chen E, et al (2000). Loss of PTEN facilitates HIF-1-mediated gene expression. Genes Dev, 14:391-396. 10691731 PMC316386

[45] Seok JK, Lee SH, Kim MJ, Lee YM (2014). MicroRNA-382 induced by HIF-1α is an angiogenic miR targeting the tumor suppressor phosphatase and tensin homolog. Nucleic Acids Res, 42:8062-8072. 24914051 10.1093/nar/gku515 PMC4081109

[46] Dodd KM, Yang J, Shen MH, Sampson JR, Tee AR (2015). mTORC1 drives HIF-1α and VEGF-A signalling via multiple mechanisms involving 4E-BP1, S6K1 and STAT3. Oncogene, 34:2239-2250. 24931163 10.1038/onc.2014.164 PMC4172452

[47] Treins C, Murdaca J, Van Obberghen E, Giorgetti-Peraldi S (2006). AMPK activation inhibits the expression of HIF-1α induced by insulin and IGF-1. Biochem Biophys Res Commun, 342:1197-1202. 16516166 10.1016/j.bbrc.2006.02.088

[48] Richard DE, Berra E, Gothie E, Roux D, Pouyssegur J (1999). p42/p44 mitogen-activated protein kinases phosphorylate hypoxia-inducible factor 1α (HIF-1α) and enhance the transcriptional activity of HIF-1. J Biol Chem, 274:32631-32637. 10551817 10.1074/jbc.274.46.32631

[49] Flügel D, Görlach A, Michiels C, Kietzmann T (2007). Glycogen synthase kinase 3 phosphorylates hypoxia-inducible factor 1α and mediates its destabilization in a VHL-independent manner. Mol Cell Biol, 27:3253-3265. 17325032 10.1128/MCB.00015-07 PMC1899978

[50] Haddad JJ, Harb HL (2005). Cytokines and the regulation of hypoxia-inducible factor (HIF)-1α. Int Immunopharmacol, 5:461-483. 15683844 10.1016/j.intimp.2004.11.009

[51] Rius J, Guma M, Schachtrup C, Akassoglou K, Zinkernagel AS, Nizet V, et al (2008). NF-κB links innate immunity to the hypoxic response through transcriptional regulation of HIF-1α. Nature, 453:807-811. 18432192 10.1038/nature06905 PMC2669289

[52] Sun H, Li XB, Meng Y, Fan L, Li M, Fang J (2013). TRAF6 upregulates expression of HIF-1α and promotes tumor angiogenesis. Cancer Res, 73:4950-4959. 23722539 10.1158/0008-5472.CAN-13-0370

[53] Scharte M, Han X, Bertges DJ, Fink MP, Delude RL (2003). Cytokines induce HIF-1 DNA binding and the expression of HIF-1-dependent genes in cultured rat enterocytes. Am J Physiol Gastrointest Liver Physiol, 284:G373-G384. 12388200 10.1152/ajpgi.00076.2002

[54] Jung YJ, Isaacs JS, Lee S, Trepel J, Neckers L (2003). IL-1β-mediated up-regulation of HIF-1α via an NFκB/COX-2 pathway identifies HIF-1 as a critical link between inflammation and oncogenesis. FASEB J, 17:2115-2117. 12958148 10.1096/fj.03-0329fje

[55] Sharma V, Dixit D, Koul N, Mehta VS, Sen E (2011). Ras regulates interleukin-1β-induced HIF-1α transcriptional activity in glioblastoma. J Mol Med (Berl), 89:123-136. 20865400 10.1007/s00109-010-0683-5

[56] van Uden P, Kenneth NS, Rocha S (2008). Regulation of hypoxia-inducible factor-1α by NF-κB. Biochem J, 412:477-484. 18393939 10.1042/BJ20080476 PMC2474706

[57] Bandarra D, Biddlestone J, Mudie S, Müller HA, Rocha S (2015). HIF-1α restricts NF-κB-dependent gene expression to control innate immunity signals. Dis Model Mech, 8:169-181. 25510503 10.1242/dmm.017285 PMC4314782

[58] Jung JE, Kim HS, Lee CS, Shin YJ, Kim YN, Kang GH, et al (2008). STAT3 inhibits the degradation of HIF-1α by pVHL-mediated ubiquitination. Exp Mol Med, 40:479-485. 18985005 10.3858/emm.2008.40.5.479 PMC2679355

[59] McMahon S, Charbonneau M, Grandmont S, Richard DE, Dubois CM (2006). Transforming growth factor β1 induces hypoxia-inducible factor-1 stabilization through selective inhibition of PHD2 expression. J Biol Chem, 281:24171-24181. 16815840 10.1074/jbc.M604507200

[60] Rozen-Zvi B, Hayashida T, Hubchak SC, Hanna C, Platanias LC, Schnaper HW (2013). TGF-β/Smad3 activates mammalian target of rapamycin complex-1 to promote collagen production by increasing HIF-1α expression. Am J Physiol Renal Physiol, 305:F485-F494. 23761672 10.1152/ajprenal.00215.2013 PMC3891259

[61] Michaud MD, Robitaille GA, Gratton JP, Richard DE (2009). Sphingosine-1-phosphate: a novel nonhypoxic activator of hypoxia-inducible factor-1 in vascular cells. Arterioscler Thromb Vasc Biol, 29:902-908. 19423865 10.1161/ATVBAHA.109.185280

[62] Nizet V, Johnson RS (2009). Interdependence of hypoxic and innate immune responses. Nat Rev Immunol, 9:609-617. 19704417 10.1038/nri2607 PMC4343208

[63] Mateo J, García-Lecea M, Cadenas S, Hernandez C, Moncada S (2003). Regulation of hypoxia-inducible factor-1α by nitric oxide through mitochondria-dependent and -independent pathways. Biochem J, 376:537-544. 14531732 10.1042/BJ20031155 PMC1223794

[64] Kietzmann T, Görlach A (2005). Reactive oxygen species in the control of hypoxia-inducible factor-mediated gene expression. Semin Cell Dev Biol, 16:474-486. 15905109 10.1016/j.semcdb.2005.03.010

[65] Chua YL, Dufour E, Dassa EP, Rustin P, Jacobs HT, Taylor CT, et al (2010). Stabilization of hypoxia-inducible factor-1α protein in hypoxia occurs independently of mitochondrial reactive oxygen species production. J Biol Chem, 285:31277-31284. 20675386 10.1074/jbc.M110.158485 PMC2951202

[66] Hagen T (2012). Oxygen versus reactive oxygen in the regulation of HIF-1α: The balance tips. Biochem Res Int, 2012:436981. 23091723 10.1155/2012/436981 PMC3474226

[67] Metzen E, Zhou J, Jelkmann W, Fandrey J, Brüne B (2003). Nitric oxide impairs normoxic degradation of HIF-1α by inhibition of prolyl hydroxylases Mol Biol Cell, 14:3470-3481. 12925778 10.1091/mbc.E02-12-0791 PMC181582

[68] Hickok JR, Vasudevan D, Antholine WE, Thomas DD (2013). Nitric oxide modifies global histone methylation by inhibiting Jumonji C domain-containing demethylases. J Biol Chem, 288:16004-16015. 23546878 10.1074/jbc.M112.432294 PMC3668755

[69] Qutub AA, Popel AS (2008). Reactive oxygen species regulate hypoxia-inducible factor 1α differentially in cancer and ischemia. Mol Cell Biol, 28:5106-5119. 18559422 10.1128/MCB.00060-08 PMC2519710

[70] Geng H, Liu Q, Xue C, David LL, Beer TM, Thomas GV, et al (2012). HIF1α protein stability is increased by acetylation at lysine 709. J Biol Chem, 287:35496-35505. 22908229 10.1074/jbc.M112.400697 PMC3471753

[71] Xenaki G, Ontikatze T, Rajendran R, Stratford IJ, Dive C, Krstic-Demonacos M, et al PCAF is an HIF-1α cofactor that regulates p53 transcriptional activity in hypoxia. Oncogene, 27:5785-5796. 18574470 10.1038/onc.2008.192 PMC2664615

[72] Lim JH, Lee YM, Chun YS, Chen J, Kim JE, Park JW (2010). Sirtuin 1 modulates cellular responses to hypoxia by deacetylating hypoxia-inducible factor 1α. Mol Cell, 38:864-878. 20620956 10.1016/j.molcel.2010.05.023

[73] Chen R, Dioum EM, Hogg RT, Gerard RD, Garcia JA (2011). Hypoxia increases sirtuin 1 expression in a hypoxia-inducible factor-dependent manner. J Biol Chem, 286:13869-13878. 21345792 10.1074/jbc.M110.175414 PMC3077588

[74] Chen S, Yin C, Lao T, Liang D, He D, Wang C, et al (2015). AMPK-HDAC5 pathway facilitates nuclear accumulation of HIF-1α and functional activation of HIF-1 by deacetylating Hsp70 in the cytosol. Cell Cycle, 14:2520-2536. 26061431 10.1080/15384101.2015.1055426 PMC4614078

[75] Kato H, Tamamizu-Kato S, Shibasaki F (2004). Histone deacetylase 7 associates with hypoxia-inducible factor 1α and increases transcriptional activity. J Biol Chem, 279:41966-41974. 15280364 10.1074/jbc.M406320200

[76] Minet E, Mottet D, Michel G, Roland I, Raes M, Remacle J, et al (1999). Hypoxia-induced activation of HIF-1: role of HIF-1α-Hsp90 interaction. FEBS Lett, 460:251-256. 10544245 10.1016/s0014-5793(99)01359-9

[77] Liu YV, Baek JH, Zhang H, Diez R, Cole RN, Semenza GL (2007). RACK1 competes with HSP90 for binding to HIF-1α and is required for O2-independent and HSP90 inhibitor-induced degradation of HIF-1α. Mol Cell, 25:207-217. 17244529 10.1016/j.molcel.2007.01.001 PMC2563152

[78] Liu YV, Hubbi ME, Pan F, McDonald KR, Mansharamani M, Cole RN, et al (2007). Calcineurin promotes hypoxia-inducible factor 1α expression by dephosphorylating RACK1 and blocking RACK1 dimerization. J Biol Chem, 282:37064-37073. 17965024 10.1074/jbc.M705015200 PMC3754800

[79] Black JC, Van Rechem C, Whetstine JR (2012). Histone lysine methylation dynamics: establishment, regulation, and biological impact. Mol Cell, 48:491-507. 23200123 10.1016/j.molcel.2012.11.006 PMC3861058

[80] Cloos PA, Christensen J, Agger K, Helin K (2008). Erasing the methyl mark: histone demethylases at the center of cellular differentiation and disease. Genes Dev, 22:1115-1140. 18451103 10.1101/gad.1652908 PMC2732404

[81] Johansson C, Tumber A, Che K, Cain P, Nowak R, Gileadi C, Oppermann U (2014). The roles of Jumonji-type oxygenases in human disease. Epigenomics, 6:89-120. 24579949 10.2217/epi.13.79 PMC4233403

[82] Sanchez-Fernandez EM, Tarhonskaya H, Al-Qahtani K, Hopkinson RJ, McCullagh JS, Schofield CJ, et al (2013). Investigations on the oxygen dependence of a 2-oxoglutarate histone demethylase. Biochem J, 449:491-496. 23092293 10.1042/BJ20121155 PMC4673901

[83] Shmakova A, Batie M, Druker J, Rocha S (2014). Chromatin and oxygen sensing in the context of JmjC histone demethylases. Biochem J, 462:385-395. 25145438 10.1042/BJ20140754 PMC4147966

[84] Hancock RL, Dunne K, Walport LJ, Flashman E, Kawamura A (2015). Epigenetic regulation by histone demethylases in hypoxia. Epigenomics, doi:10.2217/EPI.15.24 25832587

[85] Pollard PJ, Loenarz C, Mole DR, McDonough MA, Gleadle JM, Schofield CJ, Ratcliffe PJ (2008). Regulation of Jumonji-domain-containing histone demethylases by hypoxia-inducible factor (HIF)-1α. Biochem J, 416:387-394. 18713068 10.1042/BJ20081238

[86] Beyer S, Kristensen MM, Jensen KS, Johansen JV, Staller P (2008). The histone demethylases JMJD1A and JMJD2B are transcriptional targets of hypoxia-inducible factor HIF. J Biol Chem, 283:36542-36552. 18984585 10.1074/jbc.M804578200 PMC2662309

[87] Krieg AJ, Rankin EB, Chan D, Razorenova O, Fernandez S, Giaccia AJ (2010). Regulation of the histone demethylase JMJD1A by hypoxia-inducible factor 1α enhances hypoxic gene expression and tumor growth. Mol Cell Biol, 30:344-353. 19858293 10.1128/MCB.00444-09 PMC2798291

[88] Sar A, Ponjevic D, Nguyen M, Box AH, Demetrick DJ (2009). Identification and characterization of demethylase JMJD1A as a gene upregulated in the human cellular response to hypoxia. Cell Tissue Res, 337:223-234. 19471969 10.1007/s00441-009-0805-y

[89] Xia X, Lemieux ME, Li W, Carroll JS, Brown M, Liu XS, et al (2009). Integrative analysis of HIF binding and transactivation reveals its role in maintaining histone methylation homeostasis. Proc Natl Acad Sci U S A, 106:4260-4265. 19255431 10.1073/pnas.0810067106 PMC2657396

[90] Yang J, Ledaki I, Turley H, Gatter KC, Montero JC, Li JL, et al (2009). Role of hypoxia-inducible factors in epigenetic regulation via histone demethylases. Ann N Y Acad Sci, 1177:185-197. 19845621 10.1111/j.1749-6632.2009.05027.x

[91] Tausendschön M, Dehne N, Brüne B (2011). Hypoxia causes epigenetic gene regulation in macrophages by attenuating Jumonji histone demethylase activity. Cytokine, 53:256-262. 21131212 10.1016/j.cyto.2010.11.002

[92] Fu L, Chen L, Yang J, Ye T, Chen Y, Fang J (2012). HIF-1α-induced histone demethylase JMJD2B contributes to the malignant phenotype of colorectal cancer cells via an epigenetic mechanism. Carcinogenesis, 33:1664-1673. 22745382 10.1093/carcin/bgs217

[93] Wellmann S, Bettkober M, Zelmer A, Seeger K, Faigle M, Eltzschig HK, et al (2008). Hypoxia upregulates the histone demethylase JMJD1A via HIF-1. Biochem Biophys Res Commun, 372:892-897. 18538129 10.1016/j.bbrc.2008.05.150

[94] Cheutin T, Cavalli G (2014). Polycomb silencing: from linear chromatin domains to 3D chromosome folding. Curr Opin Genet Dev, 25:30-37. 24434548 10.1016/j.gde.2013.11.016

[95] Lee HY, Choi K, Oh H, Park YK, Park H (2014). HIF-1-dependent induction of Jumonji domain-containing protein (JMJD) 3 under hypoxic conditions. Mol Cells, 37:43-50. 24552709 10.14348/molcells.2014.2250 PMC3907005

[96] Salminen A, Kaarniranta K, Hiltunen M, Kauppinen A (2014). Histone demethylase Jumonji D3 (JMJD3/KDM6B) at the nexus of epigenetic regulation of inflammation and the aging process. J Mol Med (Berl), 92:1035-1043. 24925089 10.1007/s00109-014-1182-x

[97] Koivunen P, Hirsilä M, Kivirikko KI, Myllyharju J (2006). The length of peptide substrates has a marked effect on hydroxylation by the hypoxia-inducible factor prolyl 4-hydroxylases. J Biol Chem, 281:28712-28720. 16885164 10.1074/jbc.M604628200

[98] Iacobas DA, Fan C, Iacobas S, Spray DC, Haddad GG (2006). Transcriptomic changes in developing kidney exposed to chronic hypoxia. Biochem Biophys Res Commun, 349:329-338. 16934745 10.1016/j.bbrc.2006.08.056

[99] Wikenheiser J, Wolfram JA, Gargesha M, Yang K, Karunamuni G, Wilson DL, et al (2009). Altered hypoxia-inducible factor-1α expression levels correlate with coronary vessel anomalies. Dev Dyn, 238:2688-2700. 19777592 10.1002/dvdy.22089 PMC3724469

[100] Chen H, Yan Y, Davidson TL, Shinkai Y, Costa M (2006). Hypoxic stress induces dimethylated histone H3 lysine 9 through histone methyltransferase G9a in mammalian cells. Cancer Res, 66:9009-9016. 16982742 10.1158/0008-5472.CAN-06-0101

[101] Benlhabib H, Mendelson CR (2011). Epigenetic regulation of surfactant protein A gene (SP-A) expression in fetal lung reveals a critical role for Suv39h methyltransferases during development and hypoxia. Mol Cell Biol, 31:1949-1958. 21402781 10.1128/MCB.01063-10 PMC3133366

[102] Liu X, Chen Z, Xu C, Leng X, Cao H, Ouyang G, et al (2015). Repression of hypoxia-inducible factor α signaling by Set7-mediated methylation. Nucleic Acids Res, 43:5081-5098. 25897119 10.1093/nar/gkv379 PMC4446437

[103] Johnson AB, Barton MC (2007). Hypoxia-induced and stress-specific changes in chromatin structure and function. Mutat Res, 618:149-162. 17292925 10.1016/j.mrfmmm.2006.10.007 PMC1924842

[104] Watson JA, Watson CJ, McCann A, Baugh J (2010). Epigenetics, the epicenter of the hypoxic response. Epigenetics, 5:293-296. 20418669 10.4161/epi.5.4.11684

[105] Perez-Perri JI, Acevedo JM, Wappner P (2011). Epigenetics: new questions on the response to hypoxia. Int J Mol Sci, 12:4705-4721. 21845106 10.3390/ijms12074705 PMC3155379

[106] Semenza GL (2011). Hypoxia-inducible factor 1: regulator of mitochondrial metabolism and mediator of ischemic preconditioning. Biochim Biophys Acta, 1813:1263-1268. 20732359 10.1016/j.bbamcr.2010.08.006 PMC3010308

[107] Storey KB (2015). Regulation of hypometabolism: insights into epigenetic controls. J Exp Biol, 218:150-159. 25568462 10.1242/jeb.106369

[108] Buffenstein R (2005). The naked mole-rat: a new long-living model for human aging research. J Gerontol A Biol Sci Med Sci, 60:1369-1377. 16339321 10.1093/gerona/60.11.1369

[109] Lorenzen JM, Martino F, Thum T (2012). Epigenetic modifications in cardiovascular disease. Basic Res Cardiol., 107:245. 22234702 10.1007/s00395-012-0245-9 PMC3329881

[110] Wang J, Yu JT, Tan MS, Jiang T, Tan L (2013). Epigenetic mechanisms in Alzheimer's disease: implications for pathogenesis and therapy. Ageing Res Rev, 12:1024-1041. 23688931 10.1016/j.arr.2013.05.003

[111] Nguyen MP, Lee S, Lee YM (2013). Epigenetic regulation of hypoxia inducible factor in diseases and therapeutics. Arch Pharm Res, 36:252-263. 23440580 10.1007/s12272-013-0058-x

[112] Cedar H, Bergman Y (2009). Linking DNA methylation and histone modification: patterns and paradigms. Nat Rev Genet, 10:295-304. 19308066 10.1038/nrg2540

[113] Estaras C, Fueyo R, Akizu N, Beltran S, Martinez-Balbas MA (2013). RNA polymerase II progression through H3K27me3-enriched gene bodies requires JMJD3 histone demethylase. Mol Biol Cell, 24:351-360. 23243002 10.1091/mbc.E12-07-0561 PMC3564542

[114] Johnson AB, Denko N, Barton MC (2008). Hypoxia induces a novel signature of chromatin modifications and global repression of transcription. Mutat Res, 640:174-179. 18294659 10.1016/j.mrfmmm.2008.01.001 PMC2346607

[115] Toyokawa G, Cho HS, Iwai Y, Yoshimatsu M, Takawa M, Hayami S, et al (2011). The histone demethylase JMJD2B plays an essential role in human carcinogenesis through positive regulation of cyclin-dependent kinase 6. Cancer Prev Res (Phila), 4:2051-2061. 21930796 10.1158/1940-6207.CAPR-11-0290

[116] Young LC, Hendzel MJ (2013). The oncogenic potential of Jumonji D2 (JMJD2/KDM4) histone demethylase overexpression. Biochem Cell Biol, 91:369-377. 24219278 10.1139/bcb-2012-0054

[117] Awwad SW, Ayoub N (2015). Overexpression of KDM4 lysine demethylases disrupts the integrity of the DNA mismatch repair pathway. Biol Open, 4:498-504. 25770186 10.1242/bio.201410991 PMC4400592

[118] Conde-Perezprina JC, Leon-Galvan MA, Konigsberg M (2012). DNA mismatch repair system: repercussions in cellular homeostasis and relationship with aging. Oxid Med Cell Longev, 2012:728430. 23213348 10.1155/2012/728430 PMC3504481

[119] Perrigue PM, Silva ME, Warden CD, Feng NL, Reid MA, Mota DJ, et al (2015). The histone demethylase jumonji coordinates cellular senescence including secretion of neural stem cell-attracting cytokines. Mol Cancer Res, 13:636-650. 25652587 10.1158/1541-7786.MCR-13-0268 PMC4844544

[120] Fähling M (2009). Surviving hypoxia by modulation of mRNA translation rate. J Cell Mol Med, 13:2770-2779. 19674191 10.1111/j.1582-4934.2009.00875.x PMC4498934

[121] Watson JA, Watson CJ, McCrohan AM, Woodfine K, Tosetto M, McDaid J, et al (2009). Generation of an epigenetic signature by chronic hypoxia in prostate cells. Hum Mol Genet, 18:3594-3604. 19584087 10.1093/hmg/ddp307

[122] Robinson CM, Neary R, Levendale A, Watson CJ, Baugh JA (2012). Hypoxia-induced DNA hypermethylation in human pulmonary fibroblasts is associated with Thy-1 promoter methylation and the development of a pro-fibrotic phenotype. Respir Res, 13:74. 22938014 10.1186/1465-9921-13-74 PMC3519562

[123] Schweizer S, Meisel A, Märschenz S (2013). Epigenetic mechanisms in cerebral ischemia. J Cereb Blood Flow Metab, 33:1335-1346. 23756691 10.1038/jcbfm.2013.93 PMC3764391

[124] Trojer P, Reinberg D (2007). Facultative heterochromatin: is there a distinctive molecular signature? Mol Cell, 28:1-13. 17936700 10.1016/j.molcel.2007.09.011

[125] Beisel C, Paro R (2011). Silencing chromatin: comparing modes and mechanisms. Nat Rev Genet, 12:123-135. 21221116 10.1038/nrg2932

[126] Margueron R, Reinberg D (2011). The Polycomb complex PRC2 and its mark in life. Nature, 469:343-349. 21248841 10.1038/nature09784 PMC3760771

[127] Zofall M, Grewal SI (2006). Swi6/HP1 recruits a JmjC domain protein to facilitate transcription of heterochromatic repeats. Mol Cell, 22:681-692. 16762840 10.1016/j.molcel.2006.05.010

[128] Lin CH, Paulson A, Abmayr SM, Workman JL (2012). HP1a targets the Drosophila KDM4A demethylase to a subset of heterochromatic genes to regulate H3K36me3 levels. PLoS One, 7:e39758. 22761891 10.1371/journal.pone.0039758 PMC3384587

[129] Sdek P, Oyama K, Angelis E, Chan SS, Schenke-Layland K, MacLellan WR (2013). Epigenetic regulation of myogenic gene expression by heterochromatin protein 1α. PLoS One, 8:e58319. 23505487 10.1371/journal.pone.0058319 PMC3594309

[130] Fodor BD, Kubicek S, Yonezawa M, O'Sullivan RJ, Sengupta R, Perez-Burgos L, et al (2006). Jmjd2b antagonizes H3K9 trimethylation at pericentric heterochromatin in mammalian cells. Genes Dev, 20:1557-1562. 16738407 10.1101/gad.388206 PMC1482475

[131] Slee RB, Steiner CM, Herbert BS, Vance GH, Hickey RJ, Schwarz T, et al (2012). Cancer-associated alteration of pericentromeric heterochromatin may contribute to chromosome instability. Oncogene, 31:3244-3253. 22081068 10.1038/onc.2011.502

[132] Li X, Liu L, Yang S, Song N, Zhou X, Gao J, et al (2014). Histone demethylase KDM5B is a key regulator of genome stability. Proc Natl Acad Sci U S A, 111:7096-7101. 24778210 10.1073/pnas.1324036111 PMC4024858

[133] Abe Y, Rozqie R, Matsumura Y, Kawamura T, Nakaki R, Tsurutani Y, et al (2015). JMJD1A is a signal-sensing scaffold that regulates acute chromatin dynamics via SWI/SNF association for thermogenesis. Nat Commun, 6:7052. 25948511 10.1038/ncomms8052 PMC4432656

[134] Riedel CG, Dowen RH, Lourenco GF, Kirienko NV, Heimbucher T, West JA, et al (2013). DAF-16 employs the chromatin remodeller SWI/SNF to promote stress resistance and longevity. Nat Cell Biol, 15:491-501. 23604319 10.1038/ncb2720 PMC3748955

[135] Mimura I, Nangaku M, Kanki Y, Tsutsumi S, Inoue T, Kohro T, et al (2012). Dynamic change of chromatin conformation in response to hypoxia enhances the expression of GLUT3 (SLC2A3) by cooperative interaction of hypoxia-inducible factor 1 and KDM3A. Mol Cell Biol, 32:3018-3032. 22645302 10.1128/MCB.06643-11 PMC3434521

[136] Crona F, Dahlberg O, Lundberg LE, Larsson J, Mannervik M (2013). Gene regulation by the lysine demethylase KDM4A in Drosophila. Dev Biol, 373:453-463. 23195220 10.1016/j.ydbio.2012.11.011

[137] Black JC, Manning AL, Van Rechem C, Kim J, Ladd B, Cho J, et al (2013). KDM4A lysine demethylase induces site-specific copy gain and rereplication of regions amplified in tumors. Cell, 154:541-555. 23871696 10.1016/j.cell.2013.06.051 PMC3832053

[138] Zheng H, Chen L, Pledger WJ, Fang J, Chen J (2014). p53 promotes repair of heterochromatin DNA by regulating JMJD2b and SUV39H1 expression. Oncogene, 33:734-744. 23376847 10.1038/onc.2013.6 PMC3912226

[139] Khoury-Haddad H, Guttmann-Raviv N, Ipenberg I, Huggins D, Jeyasekharan AD, Ayoub N (2014). PARP1-dependent recruitment of KDM4D histone demethylase to DNA damage sites promotes double-strand break repair. Proc Natl Acad Sci U S A, 111:E728-E737. 24550317 10.1073/pnas.1317585111 PMC3932863

[140] Young LC, McDonald DW, Hendzel MJ (2013). Kdm4b histone demethylase is a DNA damage response protein and confers a survival advantage following γ-irradiation. J Biol Chem, 288:21376-21388. 23744078 10.1074/jbc.M113.491514 PMC3774405

[141] Deaton AM, Bird A (2011). CpG islands and the regulation of transcription. Genes Dev, 25:1010-1022. 21576262 10.1101/gad.2037511 PMC3093116

[142] Farcas AM, Blackledge NP, Sudbery I, Long HK, McGouran JF, Rose NR, et al (2012). KDM2B links the Polycomb Repressive Complex 1 (PRC1) to recognition of CpG islands. Elife, 1:e00205. 23256043 10.7554/eLife.00205 PMC3524939

[143] Wu X, Johansen JV, Helin K (2013). Fbxl10/Kdm2b recruits polycomb repressive complex 1 to CpG islands and regulates H2A ubiquitylation. Mol Cell, 49:1134-1146. 23395003 10.1016/j.molcel.2013.01.016

[144] Blackledge NP, Farcas AM, Kondo T, King HW, McGouran JF, Hanssen LL, et al (2014). Variant PRC1 complex-dependent H2A ubiquitylation drives PRC2 recruitment and polycomb domain formation. Cell, 157:1445-1459. 24856970 10.1016/j.cell.2014.05.004 PMC4048464

[145] Kalb R, Latwiel S, Baymaz HI, Jansen PW, Müller CW, Vermeulen M, et al (2014). Histone H2A monoubiquitination promotes histone H3 methylation in Polycomb repression. Nat Struct Mol Biol, 21:569-571. 24837194 10.1038/nsmb.2833

[146] Tzatsos A, Paskaleva P, Lymperi S, Contino G, Stoykova S, Chen Z, et al (2011). Lysine-specific demethylase 2B (KDM2B)-let-7-enhancer of zester homolog 2 (EZH2) pathway regulates cell cycle progression and senescence in primary cells. J Biol Chem, 286:33061-33069. 21757686 10.1074/jbc.M111.257667 PMC3190920

[147] Frescas D, Guardavaccaro D, Bassermann F, Koyama-Nasu R, Pagano M (2007). JHDM1B/FBXL10 is a nucleolar protein that represses transcription of ribosomal RNA genes. Nature, 450:309-313. 17994099 10.1038/nature06255

[148] Tzatsos A, Pfau R, Kampranis SC, Tsichlis PN (2009). Ndy1/KDM2B immortalizes mouse embryonic fibroblasts by repressing the Ink4a/Arf locus. Proc Natl Acad Sci U S A, 106:2641-2646. 19202064 10.1073/pnas.0813139106 PMC2650317

[149] He J, Shen L, Wan M, Taranova O, Wu H, Zhang Y (2013). Kdm2b maintains murine embryonic stem cell status by recruiting PRC1 complex to CpG islands of developmental genes. Nat Cell Biol, 15:373-384. 23502314 10.1038/ncb2702 PMC4078788

[150] Liang G, He J, Zhang Y (2012). Kdm2b promotes induced pluripotent stem cell generation by facilitating gene activation early in reprogramming. Nat Cell Biol, 14:457-466. 22522173 10.1038/ncb2483 PMC3544197

[151] Li Q, Shi L, Gui B, Yu W, Wang J, Zhang D, et al (2011). Binding of the JmjC demethylase JARID1B to LSD1/NuRD suppresses angiogenesis and metastasis in breast cancer cells by repressing chemokine CCL14. Cancer Res, 71:6899-6908. 21937684 10.1158/0008-5472.CAN-11-1523

[152] Klein BJ, Piao L, Xi Y, Rincon-Arano H, Rothbart SB, Peng D, et al (2014). The histone-H3K4-specific demethylase KDM5B binds to its substrate and product through distinct PHD fingers. Cell Rep, 6:325-335. 24412361 10.1016/j.celrep.2013.12.021 PMC3918441

[153] Zhang Y, Liang J, Li Q (2014). Coordinated regulation of retinoic acid signaling pathway by KDM5B and polycomb repressive complex 2. J Cell Biochem, 115:1528-1538. 24619877 10.1002/jcb.24807

[154] Wang X, Nagl NG, Wilsker D, Van Scoy M, Pacchione S, Yaciuk P, et al (2004). Two related ARID family proteins are alternative subunits of human SWI/SNF complexes. Biochem J, 383:319-325. 15170388 10.1042/BJ20040524 PMC1134073

[155] Barrett A, Santangelo S, Tan K, Catchpole S, Roberts K, Spencer-Dene B, et al (2007). Breast cancer associated transcriptional repressor PLU-1/JARID1B interacts directly with histone deacetylases. Int J Cancer, 121:265-275. 17373667 10.1002/ijc.22673

[156] Krishnakumar R, Kraus WL (2010). PARP-1 regulates chromatin structure and transcription through a KDM5B-dependent pathway. Mol Cell, 39:736-749. 20832725 10.1016/j.molcel.2010.08.014 PMC2939044

[157] Bueno MT, Richard S (2013). SUMOylation negatively modulates target gene occupancy of the KDM5B, a histone lysine demethylase. Epigenetics, 8:1162-1175. 23970103 10.4161/epi.26112

[158] Hendriks IA, Treffers LW, Verlaan-de Vries M, Olsen JV, Vertegaal AC (2015). SUMO-2 orchestrates chromatin modifiers in response to DNA damage. Cell Rep, http://creativecommons.org/licenses/by-nc-nd/3.0. 10.1016/j.celrep.2015.02.033 PMC4514456 25772364

[159] Schmitz SU, Albert M, Malatesta M, Morey L, Johansen JV, Bak M, et al (2011). Jarid1b targets genes regulating development and is involved in neural differentiation. EMBO J, 30:4586-4600. 22020125 10.1038/emboj.2011.383 PMC3243600

[160] Albert M, Schmitz SU, Kooistra SM, Malatesta M, Morales Torres C, et al (2013). The histone demethylase Jarid1b ensures faithful mouse development by protecting developmental genes from aberrant H3K4me3. PLoS Genet, 9:e1003461. 23637629 10.1371/journal.pgen.1003461 PMC3630093

[161] Saha B, Home P, Ray S, Larson M, Paul A, Rajendran G, et al (2013). EED and KDM6B coordinate the first mammalian cell lineage commitment to ensure embryo implantation. Mol Cell Biol, 33:2691-2705. 23671187 10.1128/MCB.00069-13 PMC3700131

[162] Burgold T, Spreafico F, De Santa F, Totaro MG, Prosperini E, Natoli G, et al (2008). The histone H3 lysine 27-specific demethylase Jmjd3 is required for neural commitment. PLoS One, 3:e3034. 18716661 10.1371/journal.pone.0003034 PMC2515638

[163] Kartikasari AE, Zhou JX, Kanji MS, Chan DN, Sinha A, Grapin-Botton A, et al (2013). The histone demethylase Jmjd3 sequentially associates with the transcription factors Tbx3 and Eomes to drive endoderm differentiation. EMBO J, 32:1393-1408. 23584530 10.1038/emboj.2013.78 PMC3655467

[164] Ohtani K, Zhao C, Dobreva G, Manavski Y, Kluge B, Braun T, et al (2013). Jmjd3 controls mesodermal and cardiovascular differentiation of embryonic stem cells. Circ Res, 113:856-862. 23856522 10.1161/CIRCRESAHA.113.302035

[165] Dahle O, Kumar A, Kuehn MR (2010). Nodal signaling recruits the histone demethylase Jmjd3 to counteract polycomb-mediated repression at target genes. Sci Signal, 3:ra48. 20571128 10.1126/scisignal.2000841 PMC6953396

[166] Miller SA, Weinmann AS (2009). An essential interaction between T-box proteins and histone-modifying enzymes. Epigenetics, 4:85-88. 19384057 10.4161/epi.4.2.8111

[167] Miller SA, Weinmann AS (2010). Molecular mechanisms by which T-bet regulates T-helper cell commitment. Immunol Rev, 238:233-246. 20969596 10.1111/j.1600-065X.2010.00952.x PMC2988494

[168] Collado M, Blasco MA, Serrano M (2007). Cellular senescence in cancer and aging. Cell, 130:223-233. 17662938 10.1016/j.cell.2007.07.003

[169] Leiser SF, Kaeberlein M (2010). The hypoxia-inducible factor HIF-1 functions as both a positive and negative modulator of aging. Biol Chem, 391:1131-1137. 20707608 10.1515/BC.2010.123 PMC4018576

[170] Philipp EE, Abele D (2010). Masters of longevity: lessons from long-lived bivalves - a mini-review. Gerontology, 56:55-65. 19468199 10.1159/000221004

[171] Shams I, Avivi A, Nevo E (2004). Hypoxic stress tolerance of the blind subterranean mole rat: expression of erythropoietin and hypoxia-inducible factor 1α. Proc Natl Acad Sci U S A, 101:9698-9703. 15210955 10.1073/pnas.0403540101 PMC470738

[172] Rivard A, Berthou-Soulie L, Principe N, Kearney M, Curry C, Branellec D, et al (2000). Age-dependent defect in vascular endothelial growth factor expression is associated with reduced hypoxia-inducible factor 1 activity. J Biol Chem, 275:29643-29647. 10882714 10.1074/jbc.M001029200

[173] Ndubuizu OI, Chavez JC, LaManna JC (2009). Increased prolyl 4-hydroxylase expression and differential regulation of hypoxia-inducible factors in the aged rat brain. Am J Physiol Regul Integr Comp Physiol, 297:R158-R165. 19420289 10.1152/ajpregu.90829.2008 PMC2711700

[174] Benderro GF, Lamanna JC (2011). Hypoxia-induced angiogenesis is delayed in aging mouse brain. Brain Res, 1389:50-60. 21402058 10.1016/j.brainres.2011.03.016 PMC3082052

[175] Rohrbach S, Teichert S, Niemann B, Franke C, Katschinski DM (2008). Caloric restriction counteracts age-dependent changes in prolyl-4-hydroxylase domain (PHD) 3 expression. Biogerontology, 9:169-176. 18236168 10.1007/s10522-008-9126-x PMC2367389

[176] Demidenko ZN, Blagosklonny MV (2011). The purpose of the HIF-1/PHD feedback loop: to limit mTOR-induced HIF-1α. Cell Cycle. 10:1557-1562. 21521942 10.4161/cc.10.10.15789

[177] Finkel T, Holbrook NJ (2000). Oxidants, oxidative stress and the biology of ageing. Nature, 408:239-247. 11089981 10.1038/35041687

[178] Salminen A, Huuskonen J, Ojala J, Kauppinen A, Kaarniranta K, Suuronen T (2008). Activation of innate immunity system during aging: NF-κB signaling is the molecular culprit of inflamm-aging. Ageing Res Rev, 7:83-105. 17964225 10.1016/j.arr.2007.09.002

[179] Chung HY, Cesari M, Anton S, Marzetti E, Giovannini S, Seo AY, et al (2009). Molecular inflammation: underpinnings of aging and age-related diseases. Ageing Res Rev, 8:18-30. 18692159 10.1016/j.arr.2008.07.002 PMC3782993

[180] Grainger DJ (2007). TGF-β and atherosclerosis in man. Cardiovasc Res, 74:213-222. 17382916 10.1016/j.cardiores.2007.02.022

[181] Symons JD, Abel ED (2013). Lipotoxicity contributes to endothelial dysfunction: a focus on the contribution from ceramide. Rev Endocr Metab Disord, 14:59-68. 23292334 10.1007/s11154-012-9235-3 PMC4180664

[182] Guarente L, Kenyon C (2000). Genetic pathways that regulate ageing in model organisms. Nature, 408:255-262. 11089983 10.1038/35041700

[183] Nicholls DG (2002). Mitochondrial function and dysfunction in the cell: its relevance to aging and aging-related disease. Int J Biochem Cell Biol, 34:1372-1381. 12200032 10.1016/s1357-2725(02)00077-8

[184] Shohet RV, Garcia JA (2007). Keeping the engine primed: HIF factors as key regulators of cardiac metabolism and angiogenesis during ischemia. J Mol Med (Berl), 85:1309-1315. 18026917 10.1007/s00109-007-0279-x

[185] Arjamaa O, Nikinmaa M, Salminen A, Kaarniranta K. Regulatory role of HIF-1α in the pathogenesis of age-related macular degeneration (AMD). Ageing Res Rev, 8:349-358. 19589398 10.1016/j.arr.2009.06.002

[186] Semenza GL (2002). HIF-1 and tumor progression: pathophysiology and therapeutics. Trends Mol Med, 8:S62-S67. 11927290 10.1016/s1471-4914(02)02317-1

[187] Luo R, Zhang W, Zhao C, Zhang Y, Wu H, Jin J, et al (2015). Elevated endothelial hypoxia-inducible factor-1α contributes to glomerular injury and promotes hypertensive chronic kidney disease. Hypertension, 66:75-84. 25987665 10.1161/HYPERTENSIONAHA.115.05578 PMC4752003

[188] Watson CJ, Collier P, Tea I, Neary R, Watson JA, Robinson C, et al (2014). Hypoxia-induced epigenetic modifications are associated with cardiac tissue fibrosis and the development of a myofibroblast-like phenotype. Hum Mol Genet, 23:2176-2188. 24301681 10.1093/hmg/ddt614

[189] Halberg N, Khan T, Trujillo ME, Wernstedt-Asterholm I, Attie AD, Sherwani S, et al (2009). Hypoxia-inducible factor 1α induces fibrosis and insulin resistance in white adipose tissue. Mol Cell Biol, 29:4467-4483. 19546236 10.1128/MCB.00192-09 PMC2725728

[190] Wierda RJ, Rietveld IM, van Eggermond MC, Belien JA, van Zwet EW, Lindeman JH, et al (2015). Global histone H3 lysine 27 triple methylation levels are reduced in vessels with advanced atherosclerotic plaques. Life Sci, 129:3-9. 25445221 10.1016/j.lfs.2014.10.010

[191] Greissel A, Culmes M, Napieralski R, Wagner E, Gebhard H, Schmitt M, et al (2015). Alternation of histone and DNA methylation in human atherosclerotic carotid plaques. Thromb Haemost, 114:390-402. 25993995 10.1160/TH14-10-0852

[192] Bell JT, Tsai PC, Yang TP, Pidsley R, Nisbet J, Glass D, et al (2012). Epigenome-wide scans identify differentially methylated regions for age and age-related phenotypes in a healthy ageing population. PLoS Genet, 8:e1002629. 22532803 10.1371/journal.pgen.1002629 PMC3330116

[193] McClay JL, Aberg KA, Clark SL, Nerella S, Kumar G, Xie LY, et al (2014). A methylome-wide study of aging using massively parallel sequencing of the methyl-CpG-enriched genomic fraction from blood in over 700 subjects. Hum Mol Genet, 23:1175-1185. 24135035 10.1093/hmg/ddt511 PMC3919012

[194] Sun D, Yi SV (2015). Impacts of chromatin states and long-range genomic segments on aging and DNA methylation. PLoS One, 10:e0128517. 26091484 10.1371/journal.pone.0128517 PMC4475080

[195] Shumaker DK, Dechat T, Kohlmaier A, Adam SA, Bozovsky MR, Erdos MR, et al (2006). Mutant nuclear lamin A leads to progressive alterations of epigenetic control in premature aging. Proc Natl Acad Sci U S A, 103:8703-8708. 16738054 10.1073/pnas.0602569103 PMC1472659

[196] Heyn H, Moran S, Esteller M (2013). Aberrant DNA methylation profiles in the premature aging disorders Hutchinson-Gilford Progeria and Werner syndrome. Epigenetics, 8:28-33. 23257959 10.4161/epi.23366 PMC3549877

[197] Zhang W, Li J, Suzuki K, Qu J, Wang P, Zhou J, et al (2015). A Werner syndrome stem cell model unveils heterochromatin alterations as a driver of human aging. Science, 348:1160-1163. 25931448 10.1126/science.aaa1356 PMC4494668

[198] Villeponteau B (1997). The heterochromatin loss model of aging. Exp Gerontol, 32:383-394. 9315443 10.1016/s0531-5565(96)00155-6

[199] Larson K, Yan SJ, Tsurumi A, Liu J, Zhou J, Gaur K, et al (2012). Heterochromatin formation promotes longevity and represses ribosomal RNA synthesis. PLoS Genet, 8:e1002473. 22291607 10.1371/journal.pgen.1002473 PMC3266895

[200] Tsurumi A, Li WX (2012). Global heterochromatin loss: a unifying theory of aging? Epigenetics, 7:680-688. 22647267 10.4161/epi.20540 PMC3414389

[201] Feser J, Tyler J (2011). Chromatin structure as a mediator of aging. FEBS Lett, 585:2041-2048. 21081125 10.1016/j.febslet.2010.11.016 PMC3988783

[202] Illi B, Ciarapica R, Capogrossi MC. Chromatin methylation and cardiovascular aging. J Mol Cell Cardiol, 83:21-31. 25724723 10.1016/j.yjmcc.2015.02.011

[203] Ollikainen M, Ismail K, Gervin K, Kyllönen A, Hakkarainen A, Lundbom J, et al (2015). Genome-wide blood DNA methylation alterations at regulatory elements and heterochromatic regions in monozygotic twins discordant for obesity and liver fat. Clin Epigenetics, 7:39. 25866590 10.1186/s13148-015-0073-5 PMC4393626

[204] Osawa T, Tsuchida R, Muramatsu M, Shimamura T, Wang F, Suehiro J, et al (2013). Inhibition of histone demethylase JMJD1A improves anti-angiogenic therapy and reduces tumor-associated macrophages. Cancer Res, 73:3019-3028. 23492365 10.1158/0008-5472.CAN-12-3231

[205] Fork C, Gu L, Hitzel J, Josipovic I, Hu J, SzeKa Wong M, et al (2015). Epigenetic regulation of angiogenesis by JARID1B-induced repression of HOXA5. Arterioscler Thromb Vasc Biol, 35:1645-1652. 26023081 10.1161/ATVBAHA.115.305561

[206] Ohtani K, Vlachojannis GJ, Koyanagi M, Boeckel JN, Urbich C, Farcas R, et al (2011). Epigenetic regulation of endothelial lineage committed genes in pro-angiogenic hematopoietic and endothelial progenitor cells. Circ Res, 109:1219-1229. 21980126 10.1161/CIRCRESAHA.111.247304

[207] Funayama R, Ishikawa F (2007). Cellular senescence and chromatin structure. Chromosoma, 116:431-440. 17579878 10.1007/s00412-007-0115-7

[208] Chandra T, Ewels PA, Schoenfelder S, Furlan-Magaril M, Wingett SW, Kirschner K, et al (2015). Global reorganization of the nuclear landscape in senescent cells. Cell Rep. 10:471-483. 25640177 10.1016/j.celrep.2014.12.055 PMC4542308

[209] Campisi J (2005). Senescent cells, tumor suppression, and organismal aging: good citizens, bad neighbors. Cell, 120:513-522. 15734683 10.1016/j.cell.2005.02.003

[210] Jeyapalan JC, Sedivy JM (2008). Cellular senescence and organismal aging. Mech Ageing Dev, 129:467-474. 18502472 10.1016/j.mad.2008.04.001 PMC3297662

[211] Salminen A, Ojala J, Kaarniranta K, Haapasalo A, Hiltunen M, Soininen H (2011). Astrocytes in the aging brain express characteristics of senescence-associated secretory phenotype. Eur J Neurosci, 34:3-11. 21649759 10.1111/j.1460-9568.2011.07738.x

[212] Ovadya Y, Krizhanovsky V (2014). Senescent cells: SASPected drivers of age-related pathologies. Biogerontology, 15:627-642. 25217383 10.1007/s10522-014-9529-9

[213] Zhu Y, Armstrong JL, Tchkonia T, Kirkland JL (2014). Cellular senescence and the senescent secretory phenotype in age-related chronic diseases. Curr Opin Clin Nutr Metab Care, 17:324-328. 24848532 10.1097/MCO.0000000000000065

[214] Campisi J, Andersen JK, Kapahi P, Melov S (2011). Cellular senescence: a link between cancer and age-related degenerative disease? Semin Cancer Biol, 21:354-359. 21925603 10.1016/j.semcancer.2011.09.001 PMC3230665

[215] Gil J, Peters G (2006). Regulation of the INK4b-ARF-INK4a tumour suppressor locus: all for one or one for all. Nat Rev Mol Cell Biol, 7:667-677. 16921403 10.1038/nrm1987

[216] Popov N, Gil J (2010). Epigenetic regulation of the INK4b-ARF-INK4a locus: in sickness and in health. Epigenetics, 5:685-690. 20716961 10.4161/epi.5.8.12996 PMC3052884

[217] Aguilo F, Zhou MM, Walsh MJ (2011). Long noncoding RNA, polycomb, and the ghosts haunting INK4b-ARF-INK4a expression. Cancer Res, 71:5365-5369. 21828241 10.1158/0008-5472.CAN-10-4379 PMC3339196

[218] Agger K, Cloos PA, Rudkjaer L, Williams K, Andersen G, Christensen J, et al (2009). The H3K27me3 demethylase JMJD3 contributes to the activation of the INK4A-ARF locus in response to oncogene- and stress-induced senescence. Genes Dev, 23:1171-1176. 19451217 10.1101/gad.510809 PMC2685535

[219] Agherbi H, Gaussmann-Wenger A, Verthuy C, Chasson L, Serrano M, Djabali M (2009). Polycomb mediated epigenetic silencing and replication timing at the INK4a/ARF locus during senescence. PLoS One., 4:e5622. 19462008 10.1371/journal.pone.0005622 PMC2680618

[220] Barradas M, Anderton E, Acosta JC, Li S, Banito A, Rodriguez-Niedenführ M, et al (2009). Histone demethylase JMJD3 contributes to epigenetic control of INK4a/ARF by oncogenic RAS. Genes Dev, 23:1177-1182. 19451218 10.1101/gad.511109 PMC2685533

[221] Pasmant E, Sabbagh A, Vidaud M, Bieche I (2011). ANRIL, a long, noncoding RNA, is an unexpected major hotspot in GWAS. FASEB J, 25:444-448. 20956613 10.1096/fj.10-172452

[222] Nijwening JH, Geutjes EJ, Bernards R, Beijersbergen RL (2011). The histone demethylase Jarid1b (Kdm5b) is a novel component of the Rb pathway and associates with E2f-target genes in MEFs during senescence. PLoS One, 6:e25235. 21980403 10.1371/journal.pone.0025235 PMC3181323

[223] Chicas A, Kapoor A, Wang X, Aksoy O, Evertts AG, Zhang MQ, et al (2012). H3K4 demethylation by Jarid1a and Jarid1b contributes to retinoblastoma-mediated gene silencing during cellular senescence. Proc Natl Acad Sci U S A, 109:8971-8976. 22615382 10.1073/pnas.1119836109 PMC3384172

[224] Narita M, Nunez S, Heard E, Narita M, Lin AW, Hearn SA, et al (2003). Rb-mediated heterochromatin formation and silencing of E2F target genes during cellular senescence. Cell, 113:703-716. 12809602 10.1016/s0092-8674(03)00401-x

[225] Leontieva OV, Natarajan V, Demidenko ZN, Burdelya LG, Gudkov AV, Blagosklonny MV (2012). Hypoxia suppresses conversion from proliferative arrest to cellular senescence. Proc Natl Acad Sci U S A, 109:13314-13318. 22847439 10.1073/pnas.1205690109 PMC3421205

[226] Kilic Eren M, Tabor V (2014). The role of hypoxia inducible factor-1α in bypassing oncogene-induced senescence. PLoS One, 9:e101064. 24984035 10.1371/journal.pone.0101064 PMC4077769

[227] Leontieva OV, Blagosklonny MV (2012). Hypoxia and gerosuppression: the mTOR saga continues. Cell Cycle, 11:3926-3931. 22987149 10.4161/cc.21908 PMC3507487

[228] Ene CI, Edwards L, Riddick G, Baysan M, Woolard K, Kotliarova S, et al (2012). Histone demethylase Jumonji D3 (JMJD3) as a tumor suppressor by regulating p53 protein nuclear stabilization. PLoS One, 7:e51407. 23236496 10.1371/journal.pone.0051407 PMC3517524

[229] Palazon A, Goldrath AW, Nizet V, Johnson RS (2014). HIF transcription factors, inflammation, and immunity. Immunity, 41:518-528. 25367569 10.1016/j.immuni.2014.09.008 PMC4346319

[230] De Santa F, Narang V, Yap ZH, Tusi BK, Burgold T, Austenaa L, et al (2009). Jmjd3 contributes to the control of gene expression in LPS-activated macrophages. EMBO J, 28:3341-3352. 19779457 10.1038/emboj.2009.271 PMC2752025

[231] Das ND, Jung KH, Choi MR, Yoon HS, Kim SH, Chai YG (2012). Gene networking and inflammatory pathway analysis in a JMJD3 knockdown human monocytic cell line. Cell Biochem Funct, 30:224-232. 22252741 10.1002/cbf.1839

[232] Salminen A, Kauppinen A, Kaarniranta K (2012). Emerging role of NF-κB signaling in the induction of senescence-associated secretory phenotype (SASP). Cell Signal, 24:835-845. 22182507 10.1016/j.cellsig.2011.12.006

[233] Lopez-Rovira T, Chalaux E, Rosa JL, Bartrons R, Ventura F (2000). Interaction and functional cooperation of NF-κB with Smads. Transcriptional regulation of the junB promoter. J Biol Chem, 275:28937-28946. 10874048 10.1074/jbc.M909923199

[234] Lan HY, Chung AC (2012). TGF-β/Smad signaling in kidney disease. Semin Nephrol, 32:236-243. 22835454 10.1016/j.semnephrol.2012.04.002

[235] Zhao L, Zhang Y, Gao Y, Geng P, Lu Y, Liu X, et al (2015). JMJD3 promotes SAHF formation in senescent WI38 cells by triggering an interplay between demethylation and phosphorylation of RB protein. Cell Death Differ, 22:1630-1640. 25698448 10.1038/cdd.2015.6 PMC4563788

[236] Higgins DF, Kimura K, Bernhardt WM, Shrimanker N, Akai Y, Hohenstein B, et al (2007). Hypoxia promotes fibrogenesis in vivo via HIF-1 stimulation of epithelial-to-mesenchymal transition. J Clin Invest, 117:3810-3820. 18037992 10.1172/JCI30487 PMC2082142

[237] Haase VH (2009). Oxygen regulates epithelial-to-mesenchymal transition: insights into molecular mechanisms and relevance to disease. Kidney Int, 76:492-499. 19536078 10.1038/ki.2009.222 PMC3623274

[238] Nieto MA (2011). The ins and outs of the epithelial to mesenchymal transition in health and disease. Annu Rev Cell Dev Biol, 27:347-376. 21740232 10.1146/annurev-cellbio-092910-154036

[239] Higgins DF, Kimura K, Iwano M, Haase VH (2008). Hypoxia-inducible factor signaling in the development of tissue fibrosis. Cell Cycle, 7:1128-1132. 18418042 10.4161/cc.7.9.5804 PMC3784650

[240] Pohlers D, Brenmoehl J, Löffler I, Müller CK, Leipner C, Schultze-Mosgau S, et al (2009). TGF-β and fibrosis in different organs - molecular pathway imprints. Biochim Biophys Acta, 1792:746-756. 19539753 10.1016/j.bbadis.2009.06.004

[241] Wu CY, Tsai YP, Wu MZ, Teng SC, Wu KJ (2012). Epigenetic reprogramming and post-transcriptional regulation during the epithelial-mesenchymal transition. Trends Genet, 28:454-463. 22717049 10.1016/j.tig.2012.05.005

[242] Stadler SC, Allis CD (2012). Linking epithelial-to-mesenchymal-transition and epigenetic modifications. Semin Cancer Biol, 22:404-410. 22706095 10.1016/j.semcancer.2012.06.007 PMC3445725

[243] Ramadoss S, Chen X, Wang CY (2012). Histone demethylase KDM6B promotes epithelial-mesenchymal transition. J Biol Chem, 287:44508-44517. 23152497 10.1074/jbc.M112.424903 PMC3531764

[244] Sun S, Sun W, Xia L, Liu L, Du R, He L, et al (2014). The T-box transcription factor Brachyury promotes renal interstitial fibrosis by repressing E-cadherin expression. Cell Commun Signal, 12:76. 25433496 10.1186/s12964-014-0076-4 PMC4261244

